# Repurposing clinically approved cephalosporins for tuberculosis therapy

**DOI:** 10.1038/srep34293

**Published:** 2016-09-28

**Authors:** Santiago Ramón-García, Rubén González del Río, Angel Santos Villarejo, Gaye D. Sweet, Fraser Cunningham, David Barros, Lluís Ballell, Alfonso Mendoza-Losana, Santiago Ferrer-Bazaga, Charles J. Thompson

**Affiliations:** 1 Department of Microbiology and Immunology, Centre for Tuberculosis Research, Life Sciences Centre, University of British Columbia, Vancouver, B.C. V6T 1Z3, Canada; 2 GlaxoSmithKline-Diseases of the Developing World, Tres Cantos, Madrid, Spain

## Abstract

While modern cephalosporins developed for broad spectrum antibacterial activities have never been pursued for tuberculosis (TB) therapy, we identified first generation cephalosporins having clinically relevant inhibitory concentrations, both alone and in synergistic drug combinations. Common chemical patterns required for activity against *Mycobacterium tuberculosis* were identified using structure-activity relationships (SAR) studies. Numerous cephalosporins were synergistic with rifampicin, the cornerstone drug for TB therapy, and ethambutol, a first-line anti-TB drug. Synergy was observed even under intracellular growth conditions where beta-lactams typically have limited activities. Cephalosporins and rifampicin were 4- to 64-fold more active in combination than either drug alone; however, limited synergy was observed with rifapentine or rifabutin. Clavulanate was a key synergistic partner in triple combinations. Cephalosporins (and other beta-lactams) together with clavulanate rescued the activity of rifampicin against a rifampicin resistant strain. Synergy was not due exclusively to increased rifampicin accumulation within the mycobacterial cells. Cephalosporins were also synergistic with new anti-TB drugs such as bedaquiline and delamanid. Studies will be needed to validate their *in vivo* activities. However, the fact that cephalosporins are orally bioavailable with good safety profiles, together with their anti-mycobacterial activities reported here, suggest that they could be repurposed within new combinatorial TB therapies.

Tuberculosis (TB), caused by *Mycobacterium tuberculosis* (*Mtb*), is presently the most deadly infectious disease worldwide. Standard TB therapy is lengthy (typically 6 months) and has very unpleasant side effects. Poor adherence to the therapy can result in the development of drug resistant forms of the disease. Patients infected with multi-drug resistant (MDR) or extensively drug resistant (XDR) strains must undergo treatments that are even longer (up to 24 months) and associated with severe side effects. Even after such prolonged and onerous therapies, only 30–50% of patients have positive treatment outcomes^1^. New treatments are urgently needed to shorten the duration of the standard treatment and for MDR and XDR-TB therapy.

Traditionally, the discovery of new antibacterial therapies has focused on finding new compounds having novel targets^2^. This extremely expensive and time-consuming strategy is currently not a practical option for most large pharmaceutical and biotech companies. The latest studies indicate that the cost of developing a new drug has soared to $2.6 billion^3^. This problem is acute in the field of TB therapy since the intrinsic resistance systems of *Mtb* make most antibiotics ineffective^4^. In other therapeutic areas, pharmaceutical companies are exploring new applications for existing drugs (repurposing) to reduce the cost of drug development^5^. We have previously demonstrated that combinatorial drug therapy, traditionally designed to avoid emergence of drug resistant *Mtb* strains, might also be employed to increase the efficacies of available antibiotics, allowing them to be repurposed for TB therapy within synergistic combinations^6^. Following this approach we aimed to improve the anti-TB activity of rifampicin, the cornerstone drug for TB therapy.

Today’s TB treatment guidelines, established in 1971, define a maximal dose of rifampicin guided largely by cost and toxicity concerns rather than maximizing antibacterial activity^7^. Recent clinical evidence demonstrating a direct relation between increased dose and therapeutic efficacy strongly suggests rifampicin is not currently administered at an optimal dose^8^,^9^. Furthermore, laboratory studies of rifampicin at higher concentrations demonstrated dose-dependent bactericidal and sterilizing activities of rifampicin against actively growing and persister cells^10^. Mutations in the *rpoB* gene are the primary cause of resistance to rifampicin in clinical isolates leading to treatment failure^11^. Studies by Louw *et al*. showed that efflux pump inhibitors could potentiate the activity of rifampicin against MDR strains by increasing its intracellular concentration^12^. Together, these reports suggest strongly that if rifampicin activity could be increased by co-administration of a synergistic partner, therapy of drug sensitive TB disease might be shortened and the prevalence of drug resistant clinical strains reduced. Increased activity against rifampicin resistant strains might also allow the re-introduction of rifampicin for therapy of MDR- and XDR-TB. This would be a major advance in managing the rising numbers of TB cases that are virtually untreatable.

In pursuit of this vision, we screened an *in-house* library of ca. 600 commercially available antibiotics (the Sweet library^13^), and found that the cephalosporins had strong synergies with rifampicin. While the activities of some beta-lactams against *Mtb* have been reported in recent years^14^,^15^,^16^, cephalosporins have never been pursued for TB therapy^17^,^18^. Here we report a comprehensive study of the activities of commercially available cephalosporins against *Mtb* alone and in combination with synergistic partners. We compared these activities with faropenem, meropenem and amoxicillin plus clavulanate (beta-lactams currently proposed for TB therapy) as a guide for further pre-clinical development.

## Material and Methods

### Bacterial strains, general growth conditions and reagents

Compounds and *Mycobacterium* strains used in this study are listed in Table S1. Mycobacteria were routinely propagated at 37 °C in Middlebrook 7H9 broth (Difco) supplemented with 10% Middlebrook albumin-dextrose-catalase (ADC)(Difco), 0.2% glycerol and 0.05% (vol/vol) tyloxapol or on Middlebrook 7H10 agar plates (Difco) supplemented with 10% (vol/vol) oleic acid-albumin-dextrose-catalase (OADC)(Difco). Hygromycin B was added to the medium (50 μg/mL) to ensure plasmid maintenance when propagating the *Mtb* H37Rv-Luc strain. This strain constitutively expresses the luciferase *luc* gene from *Photinus pyralis* (GenBank Accession Number M15077) cloned in a mycobacterial shuttle plasmid derived from pACE-1^19^.

### Drug susceptibility assays

Stock solutions of compounds used in this study were always prepared fresh on the same day of plate inoculation. For the 96-well plate format, stock solutions of compounds were prepared in their optimal solvent and manually added to polystyrene plates in two-fold serial dilutions. For 384-well plate format, compounds were dissolved in DMSO and dispensed using an HP D3000 Digital Dispenser and HP T8 Dispenserhead Cassettes (Ref No. CV081A). Susceptibility assays were performed in both extracellular and intracellular conditions. (*i*) *Extracellular.* Minimal Inhibitory Concentrations (MIC) were determined in 7H9-based broth medium. This was supplemented with 0.2% glycerol and 10% ADC without tyloxapol. When needed, other carbon sources were added to the 7H9-based broth medium. Compound efficacy on cholesterol as the sole carbon source was performed as follows: cholesterol was brought into solution (100 mM) by frequent vortexing and heating at 65 °C in ethanol-tyloxapol (1:1 v/v). A 1/1,000 dilution was then added to 7H9-based broth medium to give a final concentration of 0.1 mM cholesterol. Mycobacterial cells were grown to an OD_600_ = 0.5–0.8 and stocks were frozen at −80 °C. Upon thawing, cells were briefly sonicated and diluted in assay medium to a final concentration of 10^5^ cells/mL (OD_600_ = 0.00125) for regular assays or 10^6^ cells/mL (OD_600_ = 0.0125) for cholesterol assays. MTT [3-(4,5-dimethylthiazol-2-yl)-2,5-diphenyl tetrazolium bromide] was used as the bacterial growth indicator for *M. bovis* BCG and *Mtb*^20^. The Bright-Glo™ Luciferase Assay System (Promega, Madison, WI) was used as cell growth indicator for the *Mtb* H37Rv-Luc strain. Luminescence was measured in an Envision Multilabel Plate Reader (PerkinElmer) using the opaque 384-plate Ultra Sensitive luminescence mode, with a measurement time of 50 ms per well. Plates were incubated for 5 and 7 days before measurement of ATP production or MTT to formazan conversion, respectively^6^. The lowest concentration of drug that inhibited 90% of MTT conversion or ATP production compared to internal control wells with no drug added was used to define MIC values (IC_90_). (*ii*) *Intracellular.* Our previously described *ex-vivo* checkerboard assay^6^ was optimized based on the protocol developed by Sorrentino *et al*.^19^. Briefly, frozen stocks of macrophage THP1 cells (ATCC TIB-202) were thawed in RPMI-1640 medium (Sigma) supplemented with 10% fetal bovine serum (Gibco), 2 mM L-glutamine (Sigma) and 1 mM sodium pyruvate (Sigma). THP1 cells were passaged only 5 times and maintained without antibiotics between 2–10 × 10^5^ cells/mL at 37 °C in a humidified, 5% CO_2_ atmosphere. THP1 cells (3 × 10^8^) were simultaneously differentiated with phorbol myristate acetate (PMA, 40 ng/mL, Sigma) and infected for 4 hours at a multiplicity of infection (MOI) of 1:1 with a single cell suspension of *Mtb* H37Rv-Luc cells. After incubation, infected cells were washed four times to remove extracellular bacilli and resuspended in fresh RPMI medium. Infected cells were finally resuspended (2 × 10^5^ cells/mL) in RPMI medium supplemented with 10% fetal bovine serum (Hyclone), 2 mM L-glutamine and pyruvate and dispensed in white, flat bottom 384-well plates (Greiner) at a concentration of ca. 10,000 cells per well in a final volume of 50 μL (max. 0.5% DMSO). Plates were incubated for 5 days under 5% CO_2_ atmosphere, 37 °C, 80% relative humidity before growth assessment using the Bright-Glo Luciferase Assay System (Promega, Madison, WI) as above described. Internal wells containing drug-free medium with and without infected macrophages established maximum and minimal light production, respectively. A 90% reduction in light production was considered growth inhibition. The macrophage checkerboard data was processed as described below. Every drug or drug combination was assayed in at least three independent experiments. (*iii*) *Macrophage toxicity assay.* THP1 cells were processed as described above, but not infected, and incubated in the presence of serial dilution of the compounds for 5 days. The CellTiter-Glo Luminescent Cell Viability Assay (Promega, Madison, WI) was used to determine the viability of the macrophages; the 50% inhibitory concentration (IC_50_) was calculated relative to that for untreated cells. The human biological samples were sourced ethically and used in this study according to the terms of the informed consent.

### Semi-High Throughput Synergy Screen (sHTSS) – Primary assay

A liquid version of our previously described HTSS methodology in solid format^6^ was developed as the primary assay to identify compounds that enhanced the activity of rifampicin and ethambutol (*primary compounds*) against *Mycobacterium.* Primary compounds were screened for synergistic interactions against our *in-house* assembled compound library (Sweet library; *secondary compounds*) that included the majority of commercially available antibiotics targeting DNA, RNA, protein, cell envelope synthesis, or essential metabolic conversions, as well as other physiologically active compounds^13^. The Sweet library contains ca. 600 compounds most with unknown antimicrobial activities against mycobacteria. In order to cover the wider concentration range possible in a single run, sets of secondary compounds from the Sweet library (5 mM stock solution) were dispensed in three replicate 96-well plates [containing none, 1/8xMIC and 1/4xMIC concentrations of the primary compound (MIC_RIF_ = 0.03 μg/mL; MIC_EMB_ = 2 μg/mL)] at a maximum final concentration of 100 μM. Four-fold serial dilutions were performed to a lowest concentration of 0.006 μM. *M. bovis* BCG cells were resuspended in 7H9 broth supplemented with 10% ADS [Composition per 1 L: 9.5 g NaCl (Sigma), 50 g Bovine Serum Albumin (Sigma), 20 g D-glucose (Bio Basic Inc.) and 2% glycerol], added to every plate and incubated until analyses were performed as described above (Figure S1A).

### Checkerboard synergy assay – Secondary assay

Drug interactions identified in our primary assay (sHTSS) against *M. bovis* BCG were directly validated against *Mtb* strains and clinical isolates. Synergistic interactions were analyzed both in extracellular (7H9 broth supplemented with different carbon sources) and intracellular (THP1 infected cells) conditions. Drug activity was determined in 96-well plate format using the MTT or ATP assay, as described above. The fractional inhibitory concentration (FIC) for each compound was calculated as follows: FIC_A_ = (MIC of compound A in the presence of compound B)/(MIC of compound A alone). Similarly, the FIC for compound B (and C in triple combinations) was calculated. The FIC Index (FICI) was calculated as: FICI = [FIC_A_ + FIC_B_ (+FIC_c_)]. Synergy was defined by FICI values ≤0.5, antagonism by FICI values > 4.0, and no interaction by FICI values from 0.5 to 4.0^6^ (Figure S1B).

### *Mtb* kill-kinetics – Tertiary assay

Frozen stocks of *Mtb* were inoculated in roller bottles containing 7H9 broth supplemented with glycerol and ADC without tyloxapol to a cell density of 10^5^ cells/mL. Cultures were incubated at 37 °C for three days to allow for bacterial recovery and exponential growth. These were used to inoculate 10-mL cultures growing in 25 cm^2^ tissue culture flasks and drugs were added at the designated concentration and combinations. At every time point, cultures were thoroughly mixed and samples (100 μL) sonicated in a Sonics Vibra Cell, model VC 750, 750 W, 2 kHz, coupled to a horn cup, model CV334 set at 30 seconds, Amp 1, 45%. Samples were then 10-fold serially dilute in 1x PBS buffer with 0.1% tyloxapol and 100 μL plated on 7H10 agar plates supplemented with 10% OADC. Agar plates were incubated at 37 °C for 14 days and CFUs visualized under 10x magnification. This technique allowed accurate counting of single colonies that would eventually grow into a bigger single colony. Plates were checked again after 3 and 4 weeks of incubation to count late growers. For kill-kinetic studies under non-replicating conditions, cells were grown in roller bottles at 37 °C for 60 days before culture split, drug addition and processing as described above.

### Rifampicin and rifabutin intracellular accumulation assay

*M. bovis* BCG cultures were grown in roller bottles containing standard 7H9 media without tyloxapol to an OD_600_ = ~1.0. Cells were then diluted to OD_600_ = ~0.25 and allowed to grow for 24 hours. Pre-treatment with antibiotics was then started at their respective MIC concentrations. After overnight incubation, cells were concentrated to a final assay OD_600_ = ~15.0 in a final volume of 10 mL. Cells were incubated at 37 °C (or 4 °C) and the accumulation assay started by the addition of the rifamycin (pre-treatment antibiotic concentrations were maintained throughout the accumulation assay). At defined time points, 500 μL aliquots were diluted in 1 mL of cold PBS buffer supplemented with 0.05% tyloxapol. Cells were then washed three times to remove extracellular rifamycin and then disrupted in a FastPrep FP120 beadbeater using 0.1 mm silica beads with three 30 sec cycles at maximum speed. Cell debris was pelleted and supernatant recovered for rifamycin analysis as below described. CFUs were also determined to correlate intracellular levels of rifamycins with actual cell numbers in the sample.

### Analytical methods

Ultra-Performance Liquid Chromatography-Mass Spectrometry/Mass Spectrometry Analysis (UPLC-MS/MS) was used to quantify the amount of rifampicin, rifabutin and cephradine. The UPLC-MS/MS system consisted of an Acquity UPLC series (Waters Corporation, Madison, USA) coupled with a Sciex API 4000 instrument (AB Sciex, Toronto, Canada). Twenty microliters of every sample were added to 180 μL of protein precipitant buffer (acetonitrile /methanol 80:20 v/v) and filtered through a 0.45 μ pore size filter. Samples were then loaded into an Acquity UPLC HSS T3 50 × 2.1 mm, 1.8 μm column (Waters Corporation, Madison, USA) and eluted at an average flow rate of 0.4 mL/min. The MS/MS system was operated in MRM mode (823.5/791.3 transition for rifampicin, 847.5/815.5 for rifabutin and 350.1/158.0 for cephradine in positive ion mode). For the accumulation assay, rifampicin and rifabutin were co-eluted using an organic phase (A) of 100% acetonitrile and aqueous phase (B) of 10 mM ammonium formate and 0.1% formic acid. For the stability assays in 7H9 medium, rifampicin and cephradine were co-eluted using an organic phase (A) of 100% acetonitrile and an aqueous phase (B) of 0.1% heptafluorobutyric acid. The following elution protocol was applied: first, an initial constant gradient to 95% of B (5% of A) during 0.2 min; then, a constant gradient to 5% of B (95% of A) during 1.0 min that was held constant for a further 1.5 min. Finally, the concentration of B was gradually increased to 95% (5% of A) during 2.0 min. The actual concentration of the compounds was extrapolated from a calibration curve (1–50,000 ng/mL range) of rifampicin, rifabutin and cephradine in culture media.

## Results

### Synergy screens identified cephalosporins as the strongest synergistic partners of rifampicin

We devised a 96-well plate semi high-throughput synergy screen (sHTSS) using liquid cultures of *M. bovis* BCG to identify compounds that are synergistic with rifampicin. To maximize our hit rate, we used an *in-house* library (the Sweet library of ca. 600 compounds)^13^ that included the majority of commercially available antibiotics (more than 500). Antimicrobial activities of each compound in the Sweet library were determined alone or in the presence of our primary compound, rifampicin (1/8xMIC or 1/4xMIC) (Figure S1). Fifty hits were identified, representing about 10% of the antibiotics in the Sweet library. A secondary assay validated synergy for nine out of eleven randomly selected hit compounds, including the first-line anti-TB drug ethambutol (Table S2). When the same screen of the Sweet library was performed using ethambutol instead of rifampicin as the primary antibiotic, the rifampicin/ethambutol synergy was again identified, thereby confirming sHTSS reproducibility. In addition, not only rifampicin but also two other antibiotics (ansamycins) having related structures and targeting the RNA polymerase (RNAP) were identified as having synergy with ethambutol (Table S2). However, the most striking finding of the sHTSS was the large proportion of cell wall inhibitors identified as hits with rifampicin (14 out of 50 hits; 28%) or ethambutol (13 out of 65 hits; 20%). These mainly included the beta-lactam family of drugs and more specifically the cephalosporins (Table S2).

### Cephalosporins: Structure-activity relationship (SAR) studies reveal *Mtb*-specific features

**Figure 1:**
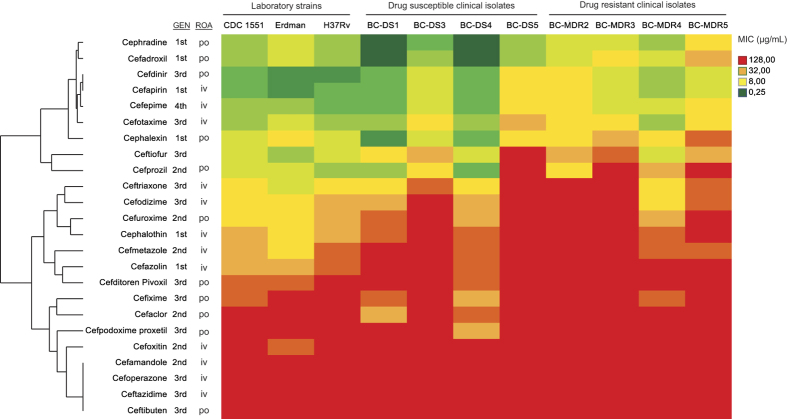
Antimicrobial activity of cephalosporins against *Mycobacterium tuberculosis* strains, including multidrug resistant clinical isolates. BC strains are clinical isolates from British Columbia, Canada. DS, Drug sensitive; MDR, multi-drug resistant. GEN, first, second and third cephalosporin generation; ROA, route of administration; po, oral; iv, intravenous.

We assembled a library of first, second, third, and forth generation cephalosporins and determined their *in vitro* activities against a panel of three standard *Mtb* laboratory strains and eight clinical isolates (four drug susceptible and four drug resistant) from British Columbia^21^. Other clinically used cell wall targeting compounds were also included in this study (Table S3). The MICs for any given cephalosporin were consistent and fell within a 4-fold range for all laboratory strains; MICs were somewhat more dispersed for the clinical isolates (probably reflecting strain genetic heterogeneity) with an overall trend of slightly decreased activity against resistant isolates. Unexpectedly, older first generation cephalosporins were generally more potent against *Mtb* than later generations (Fig. 1).

**Figure 2:**
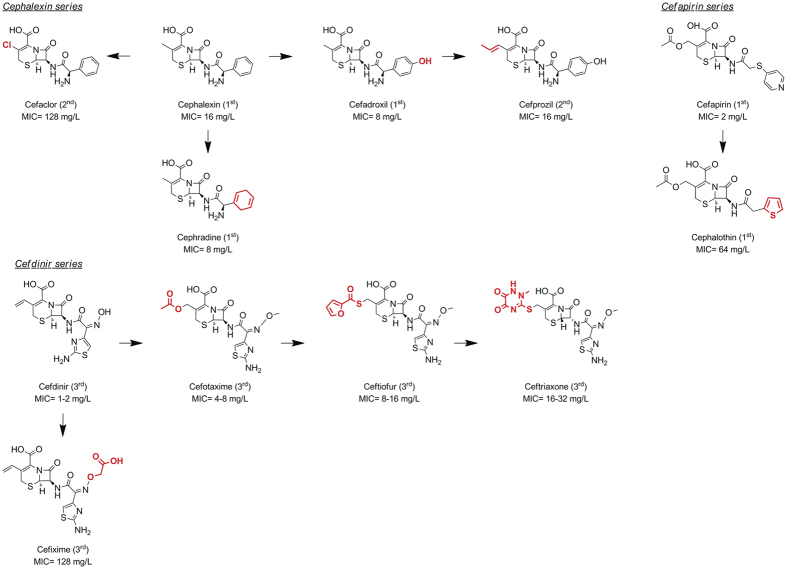
Qualitative SAR of cephalosporins against *M. tuberculosis* H37Rv. Chemical elements highlighted in red indicate modifications from the parent compounds cephalexin, cefapirin and cefdinir.

From an historical perspective, generations of cephalosporins were sequentially designed to increase broad-spectrum activity and to counteract drug resistance in a variety of non-*Mtb* pathogens^22^. These structural changes apparently altered their anti-mycobacterial activities, probably reflecting unrelated, unique features of the mycobacterial cell wall. Early reports already identified a SAR for *Mtb*, i.e., pyridyl or phenyl moieties in a side chain at the C7 position of the cephalosporin were correlated with anti-tuberculosis activity^18^. We thus inferred a qualitative SAR based on current commercially available cephalosporins. For this analysis, we created groups based on their anti-mycobacterial activity. The first group included those compounds with an activity threshold of 8 μg/mL; a second group with intermediate/moderate activity (MIC = 16–64 μg/mL); and a third group with no activity (MIC > 128 μg/mL) (Table S4). Common chemical features were identified among the active compounds, which could be further split into three representative series (Fig. 2). Analyses of both the “cephalexin and cefdinir series” indicated that a smaller lipophilic group in the C3 position of the cephem ring was beneficial for anti-mycobacterial activity; although at this position the exchange of the methyl group of cephalexin to the chloro atom of cefaclor resulted in a complete loss of activity. In addition, the comparison of cefadroxil and cefapirin (in the cephalexin and cefapirin series, respectively) indicated that the presence of a hydrogen bond acceptor in the 4 position of the right hand side (RHS) aromatic ring (either as a hetero atom within the cycle or as an extracyclic group such as a hydroxyl) was also beneficial for activity. Similarly, cefdinir (and analogs in the “cefdinir series”) also contained a hydrogen bond acceptor (amine) in the RHS of the molecule. In this series, activity was also governed by modifications in the C3 position of the cephem ring. It is interesting to note that cephradine contains a cyclohexadiene in the RHS of the molecule and it was also active. These observations indicated common generation-independent chemical patterns for specific activity against *Mtb*.

### Cephalosporin synergy with rifampicin is conserved in different media

**Figure 3:**
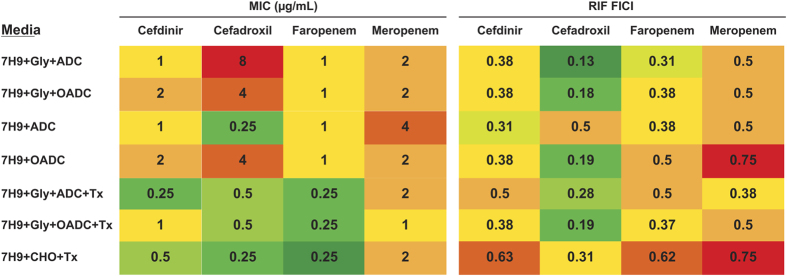
Synergistic interactions between rifampicin and selected beta-lactams against *M. tuberculosis* H37Rv cultured in different media. “RIF FICI” is the Fractional Inhibitory Concentration Index of every compound in combination with rifampicin. An FICI ≤ 0.5 indicates synergy. An FICI > 0.5 indicates no interaction. Nb, 7H9 + GLY + ADC without Tyloxapol is the standard medium used in all other experiments.

Most of the cephalosporins tested (with the exception of ceftibuten, cefuroxime, and cefditoren pivoxil) displayed synergy with rifampicin; first-generation cephalosporins cephalexin, cephradine, and cefadroxil had the strongest synergisms. The third generation cephalosporin, cefdinir, was the most active alone, but had a weaker synergistic profile (Table S3). These studies were conducted using 7H9 media supplemented with ADC and glycerol. Media conditions used by teams working on TB drug development are not standardized^23^. TB drug screening programs have found that the anti-mycobacterial activities of certain compounds are carbon source (glycerol) dependent^24^. Other supplements that alter cell envelope composition, such as oleic acid or detergents (Tween or Tyloxapol), also have effects on antibiotic activity^25^,^26^. In addition, the chemical nature of cephalosporins might affect their activity in a medium-dependent fashion^27^. To investigate possible medium-dependent activities of cephalosporins and their synergies with rifampicin, we performed synergy studies with cefadroxil and cefdinir in media containing various carbon sources or detergents. Faropenem (a penem) and meropenem (a carbapenem), two beta-lactams recently evaluated in a clinical trial^28^, were also included for comparison (Fig. 3). Cefadroxil displayed the largest medium dependent variations in anti-microbial activity but it retained the strongest synergistic profile in all the different media tested. In contrast, meropenem displayed little synergy, and in some cases a lack of interaction with rifampicin. In general, the antimicrobial activities of the cephalosporins alone and their synergistic profiles in combination with rifampicin were maintained in media based on oleic acid, cholesterol, glycerol or dextrose. Interestingly, the addition of Tyloxapol had a major potentiating effect, correlating with previous observations where alteration of the mycobacterial cell wall mycolic acid layer might allow better access of the beta-lactams to their targets in the peptidoglycan sub-layer^29^,^30^; synergy in the presence of Tyloxapol, however, remained mostly unchanged.

### The intracellular anti-mycobacterial activities of cephalosporins can be synergistically enhanced by rifampicin

**Figure 4:**
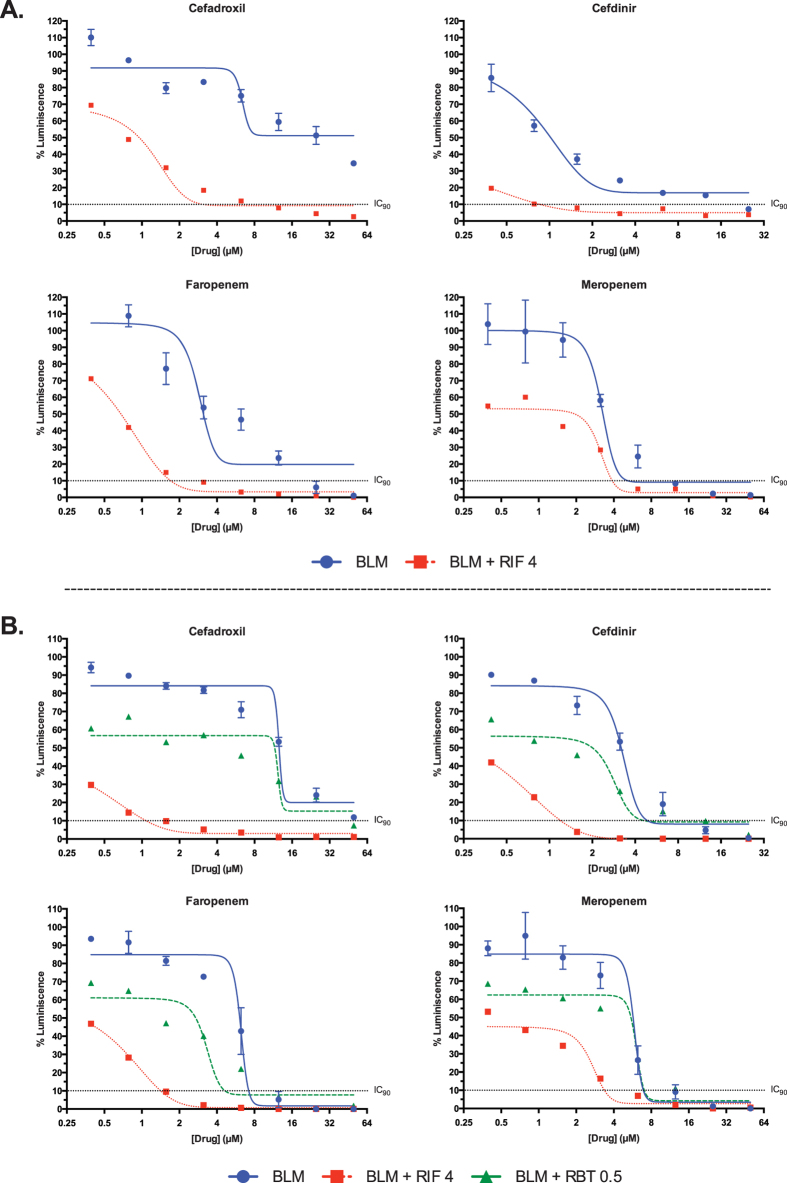
Dose response characterization of selected beta-lactam and rifamycin combinations against *M. tuberculosis*. (**A**) Intracellular (THP1) dose response curves of beta-lactams (BLM) alone and in the presence of 1/4xMIC concentrations of rifampicin against *M. tuberculosis* H37Rv-Luc. Intracellular MIC_RIF_ = 16 ng/mL. (**B**) Comparison of the synergistic effects of rifampicin and rifabutin with beta-lactams in 7H9 medium. Dose response studies of the beta-lactams in combination with sub-inhibitory concentrations of rifampicin (RIF; 2 ng/mL; 1/8xMIC) and rifabutin (RBT; 0.5 ng/mL; 1/4xMIC). The MIC of rifampicin and rifabutin were 16 and 2 ng/mL, respectively.

Although less efficient than against extracellular bacteria, cephalosporins (and beta-lactams in general) display measurable intracellular activities^31^. Similarly, we found that some cephalosporins such as cephradine, cefadroxil or cephalexin, were less active against *Mtb* inside THP1 macrophages than against extracellular bacteria (Table S3). In some cases, these limitations could be overcome using a synergistic combination. For example, the presence of rifampicin at sub-MIC concentrations made cefadroxil active against intracellular *Mtb* and the same trend was observed for cefdinir. Intracellular synergy was also observed for faropenem but not for meropenem (Fig. 4A), which displayed the weakest synergistic profile with rifampicin against intracellular and extracellular bacteria (Table S3). Despite the fact that some beta-lactams might have poor intracellular activity, these analyses demonstrated that intracellular synergies with rifampicin could compensate for their reduced activities alone.

### Synergy with cephalosporins is not a general characteristic of rifamycins

We tested the abilities of different beta-lactams to act in synergy with other representative rifamycins^32^. The dose response curves of cefadroxil, cefdinir, faropenem, and meropenem were calculated alone and in combination with rifampicin and rifabutin. Surprisingly, synergies were observed with rifampicin but not with rifabutin (Fig. 4B). We then determined the MICs of other cephalosporins, amoxicillin and cell-wall targeting compounds alone and in the presence of sub-inhibitory concentrations of the rifamycins (including rifapentine) (Table S5). While cephalosporins, ethambutol and the other beta-lactams tested interacted with rifampicin, synergy with rifapentine was consistently lower. In fact, only those drugs having an MIC reduction higher than 16-fold in the presence of rifampicin, such as cefadroxil and cephradine, displayed some interaction with rifapentine. Confirming our previous observations, no synergy was observed with rifabutin.

### Increased accumulation of rifampicin in mycobacterial cells does not fully explain its synergy with beta-lactams

**Figure 5:**
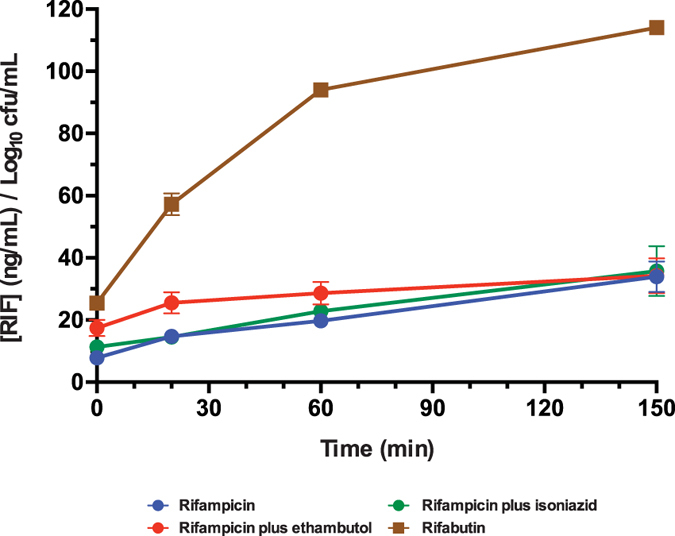
Rifamycin accumulation in *M. bovis* BGC. Rifampicin and rifabutin at 1 μg/mL were added to cell cultures incubated at 37 °C. Cells were pre-treated overnight in the presence of ethambutol (positive synergistic control; 5 μg/mL) or isoniazid (negative control; 0.25 μg/mL).

Beta-lactams that destabilize the peptidoglycan layer in the cell wall might cause increased permeability and intracellular accumulation of rifampicin, thus rationalizing synergy. To test this hypothesis, we performed rifampicin accumulation assays. Ethambutol increases rifampicin uptake^25^, presumably underlying the synergy between these two drugs (Table S3). Proof of concept experiments confirmed that rifampicin accumulation was an active process; residual accumulation was observed at low temperature (4 °C), probably associated with unspecific binding to the bacterial cell wall or passive diffusion, while a saturable active rifampicin uptake was observed at 37 °C (Figure S2A). As expected^25^, there was a ca. 2.5-fold increase in rifampicin accumulation when the cells were pre-treated with ethambutol but not when they were pre-treated with isoniazid (we found no synergy between isoniazid and rifampicin) (Fig. 5). We therefore used these two drugs in our accumulation assay as internal positive and negative controls. Pre-treatment with either cefadroxil or amoxicillin (with and without clavulanate), faropenem or meropenem had a range of effects on rifampicin accumulation. The largest effect was elicited by the combination of amoxicillin and clavulanate. Cefadroxil had little or no effect, despite the fact that it was the strongest synergistic partner and while meropenem, the weakest synergistic partner, induced increases similar to those of ethambutol (Figure S2B).

We then used rifabutin to further investigate the potential correlation between anti-mycobacterial activity and the intracellular accumulation of rifampicin. Rifabutin is more lipophilic than rifampicin; this characteristic may accelerate its penetration through the *Mtb* envelope, thus increasing its activity^32^. Indeed, rifabutin accumulated intracellularly to higher levels than rifampicin (Fig. 5); rifabutin uptake was higher than that of rifampicin (even when rifampicin uptake was increased by ethambutol or beta-lactams) (Figure S2). Rifabutin’s lack of synergism (Fig. 4B, Table S5) with beta-lactams may reflect increased rates of cell penetration that cannot be further potentiated by beta-lactam treatment. On average, the level of synergism between rifampicin and the beta-lactams allowed up to 16-fold reduction in the MIC of rifampicin, a slightly higher reduction than the MIC ratio of rifabutin/rifampicin (rifabutin’s MIC is ca. 8-fold lower than that of rifampicin). However, the observation that beta-lactams induced less than 2.5-fold increases in rifampicin accumulation indicated that they do not mimic the more efficient rifabutin pathway of entry, which allows 6-10-fold increased levels of accumulation (Fig. 5). In summary, the strong synergistic interactions between cephalosporins (and other beta-lactams) and rifampicin cannot exclusively be explained by the slight increase in the intracellular accumulation of rifampicin in the presence of some cell-wall targeting compounds since there was no correlation between the strength of the synergistic interactions and the levels of rifampicin accumulation.

### The synergistic combination of rifampicin with cephalosporins is bactericidal and sterilizing

**Figure 6:**
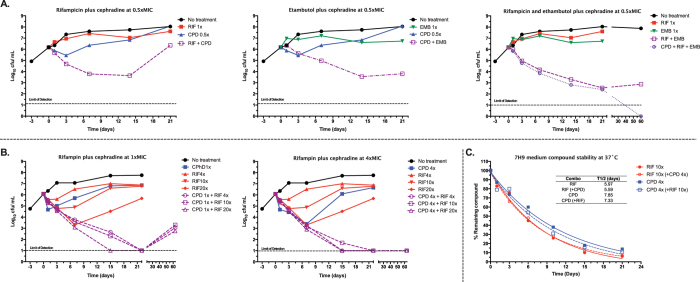
Kill kinetics of rifampicin, cephradine and ethambutol alone and in combination against *M. tuberculosis* H37Rv. (**A**) Sub-inhibitory concentrations of cephradine (0.5xMIC, 4 μg/mL) enhanced the antibacterial and sterilizing activities of rifampicin and ethambutol (no growth observed after 60 days; upper right panel). (**B**) Dose dependent sterilizing activity of cephradine. Increased concentrations of cephradine (4xMIC, 32 μg/mL) enhanced the sterilizing activity of rifampicin (no growth observed after 60 days; bottom middle panel). (**C**) The stabilities of rifampicin and cephradine alone and in combination in 7H9 media were analysed by UPLC-MS/MS. MIC values of the individual drugs are used to express drug concentrations (i.e., 1, 4, 10-fold their MIC concentrations). RIF, rifampicin; CPD, cephradine; EMB, ethambutol. MIC_RIF_ = 0.03 μg/mL; MIC_CPD_ = 8 μg/mL; MIC_EMB_ = 2 μg/mL.

Kill kinetic assays against replicating bacteria exposed to antibiotics at sub-MIC concentrations were performed to study the bactericidal and sterilizing activities of the synergistic combinations (Fig. 6). Combinations of cephradine with either rifampicin or ethambutol were able to reduce CFUs up to 3-logs within seven days. Interestingly, addition of cephradine to rifampicin and ethambutol in a triple combination at sub-MIC concentrations made these combinations sterilizing (Fig. 6A). We then increased drug concentrations to levels above MIC to determine minimal concentrations needed for sterilizing activity. Cephradine and rifampicin alone displayed dose-dependent bactericidal activity and sterilizing activity in combination (Fig. 6B). The pharmacodynamic parameters that best predict the anti-bacterial activity of the beta-lactams and rifampicin are time over MIC and the AUC over MIC^33^,^34^. We employed a static model where cultures were maintained in the same medium and drugs were only added at the beginning of the experiment. In the absence of bacteria, the half-life stability of cephradine and rifampicin in the 7H9 medium was approximately six and seven days, respectively, similar to described elsewhere^35^,^36^. Their stabilities were independent of the presence of the partner, indicating absence of direct drug-drug interactions. Degradation kinetics and remaining drug concentrations due to thermal instability matched kill kinetics and might explain the observed growth rebound (Fig. 6C). The extended killing observed with the combination in the absence of effective drug concentrations confirmed their synergistic profile (Fig. 6B). Under non-replicative conditions, cephradine lost its anti-bacterial activity together with its synergistic interaction with rifampicin (Figure S3), consistent with the specificity of some cephalosporins for actively replicating bacteria.

### Clavulanate: a key partner in triple synergistic combinations effective against rifampicin resistant *M. tuberculosis* strains

**Figure 7:**
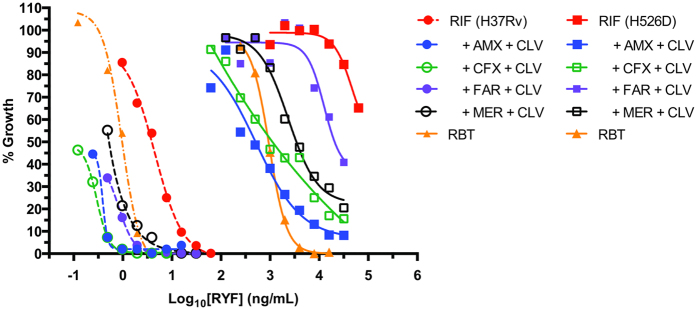
Synergistic triple combinations of rifampicin and beta-lactams. Dose response curves of rifampicin alone and in the presence of several beta-lactam combinations against *M. tuberculosis* H37Rv (rifampicin susceptible) and its rifampicin resistant derivative *M. tuberculosis* H37Rv H526D. Dose response curve of rifabutin were also included for comparison. For the drug susceptible strain, beta-lactam and clavulanate concentrations were 0.06 μg/mL and 8 μg/mL, respectively. For the rifampicin resistant strain, beta-lactam and clavulanate concentrations were 0.125 μg/mL and 5 μg/mL, respectively. AMX, amoxicillin; CFX, cefadroxil; CLV, clavulanate; FAR, faropenem; MER, meropenem; RBT, rifabutin; RIF, rifampicin.

While clavulanate had a high MIC against *Mtb* (>64 μg/mL), it was synergistic with rifampicin (Table S3). Pairwise synergies among rifampicin, beta-lactams and clavulanate suggested that the addition of clavulanate could further enhance the anti-tuberculosis activities of rifampicin/beta-lactam combinations. Therefore, we tested rifampicin in combination with beta-lactams, in the presence and absence of sub-inhibitory concentrations of clavulanate. We used the laboratory strain H37Rv and a rifampicin-resistant derivative (MIC > 64 μg/mL) resulting from a single point mutation (H526D) in the beta subunit of the RNA polymerase to limit genetic background heterogeneity. Rifampicin dose response analyses using H37Rv H526D demonstrated that the addition of a beta-lactam plus clavulanate had activities similar to rifabutin (used against MDR and XDR strains^37^). Among the four beta-lactams tested, amoxicillin had the strongest effect, followed by cefadroxil, meropenem and finally faropenem (whose synergistic activity was marginal). These combinations were even more effective than rifabutin against the parental drug susceptible H37Rv (Fig. 7). These studies not only demonstrated that beta-lactams increased rifampicin activity but also confirmed our MIC studies showing that rifampicin greatly enhanced the activities of its beta-lactam partners. Clavulanate played a critical role in these triple drug interactions, especially for beta-lactams whose activities were clavulanate-dependent such as amoxicillin and cefadroxil. Reinforcing this concept, faropenem, whose activity was not strongly affected by clavulanate, had weak effects in the triple combination (Figure S4).

### Beta-lactam inclusion in *de novo* therapies: synergy with newly developed anti-TB drugs

**Figure 8:**
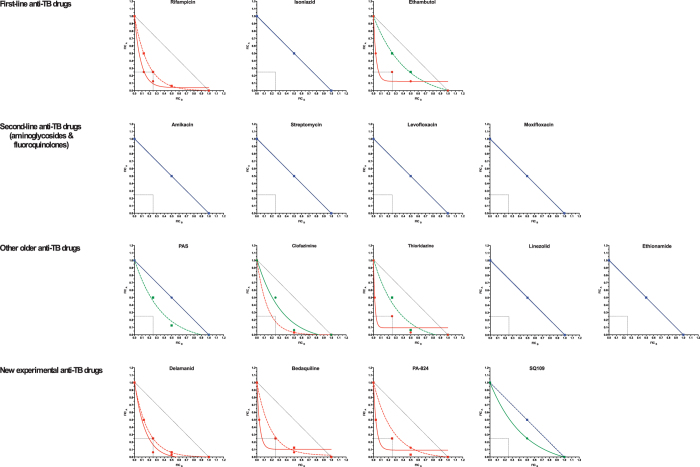
Synergistic interactions of cephradine and faropenem with a panel of anti-TB drugs against *M. tuberculosis* H37Rv. Fractional inhibitory concentrations of every drug within every pairwise combination were calculated and plotted as isobolograms to allow visual inspection of the drug interactions. A straight line indicates a non-interaction profile while curves closer to the axis origins and falling within the box indicate a synergistic profile. Isobologram curves are colour coded according to their synergistic profile. Red, synergistic interaction; Green, additive; and blue, no interaction. Cephradine, solid lines; faropenem, broken lines.

*In vitro* synergy assays with cephradine and faropenem in combination with a panel of sixteen first-line, second-line and newly developed drugs for TB therapy were performed to further explore the repurposing potential of beta-lactams (Fig. 8). As expected, synergy was observed with both rifampicin and ethambutol but not with the first-line drug isoniazid. Aminoglycosides and fluoroquinolones, well-established second-line drugs, and other drugs typically used as last resort treatment for MDR- and XDR-TB, such as PAS, ethionamide or linezolid, displayed no interaction patterns. In contrast, there were strong synergistic interactions of the beta-lactams with the newly developed anti-TB drugs bedaquiline, delamanid and PA-824 (pretomanid). Synergism was not observed with SQ-109, an ethambutol analog with an anti-bacterial mode of action that is more related to isoniazid^38^.

## Discussion

Drug repurposing is a strategy gathering momentum throughout the pharmaceutical industry, driven by the high costs of traditional drug development^3^,^5^,^39^. Drug development for neglected diseases such as TB, mainly affecting developing countries, is especially complicated due to the perception by big pharmaceutical companies that there is insufficient return on capital investment. In addition, promising pre-clinical development of new chemical entities driven by not-for-profit partnerships^40^ are faced with unexpected clinical roadblocks due to toxicities not predicted by pre-clinical models^41^. To overcome these limitations, new partnership models^42^,^43^ and drug repurposing strategies are currently being explored^6^. We previously demonstrated that clinically approved antibiotics considered to be inactive against *Mtb* might be introduced for TB therapy if administered within synergistic combinations^6^. Here we demonstrated that this approach could be similarly applied to well-established anti-TB drugs in order to increase their efficacy or reduce their toxicity. For this, we focused on rifampicin, one of the cornerstone drugs for TB therapy. We screened an *in-house* library of clinically used antibiotics and were able to find synergistic partners of rifampicin. While a wide variety of compounds were identified, including a front line TB drug (ethambutol), we focused our studies on the large pool of cephalosporin antibiotics that were strong enhancers of rifampicin activity (Table S2). Cephalosporin activities, alone and in synergistic combinations with other beta lactams, were compared to beta-lactam combinations already proposed for TB therapy (clavulanate with faropenem, meropenem, or amoxicillin), to assess their potential for TB therapy.

Beta-lactams, one of the largest groups of antibiotics available today, have a long track record of safe clinical use to treat infections caused by Gram-positive and Gram-negative bacteria^22^. Their use as anti-tuberculosis drugs has been limited by the lack of interest of pharmaceutical companies, the availability of reliable animal models^44^ and the intrinsic resistance of *Mtb* to beta-lactams. Indeed, the presence of a beta-lactamase (BlaC) able to degrade these antibiotics and the impermeability of the cell envelope led to the belief that beta-lactams were ineffective for TB therapy. As early as in the 1980s it was recognized that the combination of amoxicillin and clavulanate was active against *Mtb in vitro*^45^; however clinical efficacy in early bactericidal activity (EBA) studies was not consistently shown^46^,^47^. A renewed interest in the beta-lactams as new anti-TB drugs arose after a report demonstrated the *in vitro* activity of meropenem combined with clavulanate against XDR strains^14^. A recent study also reported synergy of carbapenems with rifampicin against *Mtb*^48^. However, although carbapenems have anecdotally been used successfully as part of salvage therapies for XDR patients, they have to be administered intravenously^16^,^49^. This would not be a practical approach in under-resourced countries, where orally delivered drugs are preferred. Recently, an EBA Phase II clinical trial has validated the promising potential of a carbapenem combined with amoxicillin and clavulanic acid for TB therapy^28^. This EBA study was able to detect activity of intravenous meropenem but not an orally available alternative, faropenem. Although faropenem has shown efficacy in combination with amoxicillin, clavulanate, and probenicid using a murine model of tuberculosis^50^, the lack of response in the EBA study could be due to the low levels of exposure and limited time above the MIC concentration. This reflects its lower bioavailability when administered as its sodium salt, the one readily available in the market. Other beta-lactams should thus be explored for TB therapy.

Among the beta-lactams, we identified the cephalosporins as the most promising group of drugs synergistic with rifampicin (Table S2). The cephalosporins have traditionally received little attention for TB treatment although they are orally available with good safety profiles, and very few drug-drug interactions (none reported with rifampicin or ethambutol). Interestingly, we found that while first-generation cephalosporins were highly potent, later generations generally had less activity (Fig. 1). This is contrary to previous studies with other bacteria where third-generation cephalosporins were more active against Gram-negative bacilli compared to first- or second-generation cephalosporins^51^. We thus assembled a qualitative SAR defining specific rules for increased activity against *Mtb* (Fig. 2); this information might allow design of a *Mtb*-specific cephalosporin. Such a compound would limit the enrichment of beta-lactam resistance genes in the gut microbiome. Increased specificity for *Mtb* would also serve to minimize gastro-intestinal side effects caused by broad-spectrum beta-lactam antibiotics. This approach, although promising and worth pursuing, would be independent of our fast-track repurposing vision of bringing new drugs for TB treatment in the shortest period possible.

The long treatment required to cure TB is, in part, rationalized by the need to eradicate drug tolerant forms of *Mtb* that reside within host cells, thus limiting antibiotic efficacy. Beta-lactams are known to be less effective against intracellular pathogens due to their inability to penetrate host membranes^52^. Here we demonstrated that the intracellular activity of the cephalosporins and faropenem could be enhanced in the presence of rifampicin, a synergistic partner that makes the bacteria sensitive to lower levels of intracellular beta-lactams (Fig. 4A).

The general concept that increases in *Mtb* permeability can determine sensitivity to both beta-lactams and rifampicin^25^ led us to explore the idea that synergy is mediated by shared effects on the cell envelope. In fact, the presence of Tyloxapol in the media, a detergent routinely added to prevent cell clumping by altering the outer envelope structure of mycobacteria, had major effects on the activities of beta-lactams or rifampicin alone, while it had very little effect on their levels of synergy (Fig. 3). This suggested that the drug permeability barriers affected by Tyloxapol are not those that determine synergy. Consistent with this notion, the levels of intracellular accumulation of rifampicin in cells pre-treated with a synergistic beta-lactam did not fully explain the strong synergism of the combination; rates of rifampicin uptake in the presence of beta-lactams were far lower than those of rifabutin, a comparable antibiotic that is more lipophilic and more active against *Mtb* (Fig. 5). The observation that synergy of rifabutin with the beta-lactams was negligible compared to rifampicin supports the hypothesis that rifabutin is able to enter the cell more rapidly, thereby bypassing the synergistic toxicities that can be elicited by various beta-lactams. In the parental strain H37Rv, rifampicin’s MIC was 8-fold higher than the MIC of rifabutin, whereas in the rifampicin resistant mutant (*Mtb* H37Rv H526D) rifampicin’s MIC was at least 64-fold higher than rifabutin (Fig. 7). The higher efficacy of rifabutin relative to rifampicin may reflect its increased accumulation (Fig. 5); this might be due to permeability or to the specificity of efflux systems for different rifamycins^12^.

In addition to permeability changes mediating synergy, there are likely to be other synergistic interactions for specific beta-lactams or rifamycins. For example, while rifampicin and rifabutin bind within the same region of the RNAP, these two rifamycins may have different effects on RNAP activity and gene expression. Rifabutin binding to RNAP differs slightly from that of rifampicin^53^ and may elicit a different transcriptional response. Indeed minor changes within this region of RNAP generated by rifampicin resistance mutations can induce different metabolic responses^54^. These different effects elicited by rifampicin or rifabutin are also reflected in their different synergies with beta-lactams. Complex transcriptional perturbations and cell wall defects, independently induced by these drugs, act together to inhibit essential bacterial functions. Importantly, cephradine and faropenem also displayed synergy with a variety of antibiotics including ethambutol, bedaquiline, delamanid, clofazimine, thioridazine, and PA824 but not with isoniazid, ethionamide, SQ109, PAS, aminoglycosides or fluoroquinolones (Fig. 8). Although elucidating the precise mode of action of these synergistic combinations is beyond the scope of this work, understanding the molecular mechanisms behind them would facilitate a rational design for combination therapies that include beta-lactams.

Dose response studies of rifampicin alone and in combination with beta-lactams under both extracellular (7H9 broth) and intracellular (*Mtb*-infected THP1 cells) growth conditions allowed us to identify the cephalosporins with the best potential (Fig. 1, Table S3). We concluded that cephradine and cefadroxil were the most potent cephalosporins, having strong synergistic activities with rifampicin. In addition, they have the best pharmacological properties, including oral bioavailability. For example, cefadroxil is commercialized under the brand name Duricef. A single 500 mg oral dose of cefadroxil would reach plasma concentrations of ca. 16 μg/mL (Table S6), well above the synergistic MIC (Table S5). One concern about the clinical use of beta-lactams is their limited exposure time due to their short pharmacokinetic half-life. Cefadroxil could be clinically effective as a single drug and its efficacy could be further increased in a synergistic combination.

Beta-lactams (including cephalosporins) are generally more active against replicating extracellular bacteria, the main bacterial pool found in TB patients that seek initial clinical assistance^55^. The combination of cephalosporins with rifampicin (and also ethambutol) was bactericidal and sterilizing against extracellular bacteria growing in liquid cultures (Fig. 6). Clavulanate was also found to be a key synergistic partner for both rifampicin and beta-lactams; this could potentially lead to re-introduction of rifampicin for MDR- and XDR-TB therapy (Fig. 7). For clinical use, clavulanate is only available in combination with amoxicillin (Augmentin). Interestingly, amoxicillin also displays synergy with some beta-lactams (Table S7), which could further potentiate their activities. However, the length of cephalosporin/beta-lactam treatment should be limited to the first weeks of therapy to avoid complications due to long exposure and unspecific targeting of the patients’ microbiota.

Our studies suggest that selected cephalosporins alone and in combination with rifampicin (or ethambutol) should be pursued as potential TB therapies. We assembled *in vitro* data that lays the foundations for future development. Our studies of cephalosporin activities using a small set of clinical isolates (4 drug susceptible and 4 MDR) suggested a trend of slightly reduced susceptibility in the MDR strains (Fig. 1). Analyzing a larger set of clinical *Mtb* isolates from different geographical regions would be needed to define actual MIC_90_ ranges of selected cephalosporins. The mouse model is the standard *in vivo* system used for testing new potential anti-TB therapies. However, this approach is of limited value for beta-lactams since their pharmacokinetics and efficacy in mice do not predict those in humans. This reflects the fact that mice express an enzyme that degrades beta lactams (renal dehydropeptidase I, DPH-I) at levels that are several orders of magnitude higher than in humans^44^,^56^. However, because beta-lactams are previously approved drugs with established dosages and known safety profiles, these combinations could be readily tested in the clinic. In fact, other beta-lactams (meropenem and faropenem in combination with amoxicillin-clavulanate) are currently being pursued as potential TB therapies^28^. We report that rifampicin activity can be also increased in combination with single cephalosporins or multiple beta-lactams, including clavulanate. Such triple synergies should be explored as ways to optimize the activity of rifampicin, the cornerstone drug in TB therapy, for treating drug sensitive and perhaps MDR and XDR-TB infections. TB drug development is evolving towards completely new therapeutic combinations able to treat all forms of the disease that would not depend on the resistance profile of the strain^57^. The activities of cephalosporins (as well as other beta-lactams) alone and in synergy with other new anti-TB drugs such as bedaquiline, pretomanid (PA-824), and delamanid, suggest additional combinations that might reduce treatment time for standard and MDR therapies.

## Additional Information

**How to cite this article**: Ramón-García, S. *et al*. Repurposing clinically approved cephalosporins for tuberculosis therapy. *Sci. Rep.* **6**, 34293; doi: 10.1038/srep34293 (2016).

## Supplementary Material

Supplementary Information
