# Supplementary material for: Repurposing clinically approved cephalosporins for tuberculosis therapy
Source: Sci Rep. 2016 Sep 28;6:34293. doi: 10.1038/srep34293 (PMC5039641; doi:10.1038/srep34293)
Supplement: Supplementary Information [file srep34293-s1.pdf]

SUPPLEMENTAL FIGURES AND TABLES

Repurposing clinically approved cephalosporins for tuberculosis therapy

Santiago Ramón-García*, Rubén González del Río, Angel Santos Villarejo, Gaye D. Sweet, Fraser Cunningham, David Barros, Lluís Ballell, Alfonso Mendoza-Losana, Santiago Ferrer-Bazaga and Charles J. Thompson

*Corresponding author. E-mail: ramon@mail.ubc.ca

Figure S1. Semi-High throughput Synergy Screen (sHTSS) (Primary assay) and checkerboard (Secondary assay) - Schematic representation. (A) 96- or 384-well plate format was used to identify hits (secondary compounds) that act in synergy with a drug of interest (primary compound, PC). Up to ten (in 96-well format) or eleven (in 384-well format) secondary compounds are assayed in a serial dilution dose response format in the absence or the presence of sub-MIC concentrations of the PC. The MIC of the PC (MIC_{PC}), previously determined, was used in the plate design to define PC sub-MIC concentrations. In addition, MIC_{PC} is internally determined for every 96- or 384-plate to assess the actual sub-MIC concentration in the test plate. Pink represents growth. Blue represents growth inhibition. Yellow crosses indicate synergistic combinations. Interactions at 1/2xMIC of PC were not considered (384-plate layout) since the primary compound exhibited strong activity alone. CT-, negative control (no cells, no drugs); CT+, positive control (cells, no drugs). (B) The checkerboard assay was used for synergistic interaction studies. In a 384-well plate format, two dimensional arrays of serial dilutions of up to four test compounds were introduced to exponentially growing cultures of *M. tuberculosis*. The fractional inhibitory concentration index (FICI) was used to determine whether paired combinations exert inhibitory effects that were more than the sum of their effects alone (FICI <0.5; synergy). Upper left and lower right represent typical appearance profiles of a synergistic combination while upper right and lower left indicate no interaction.

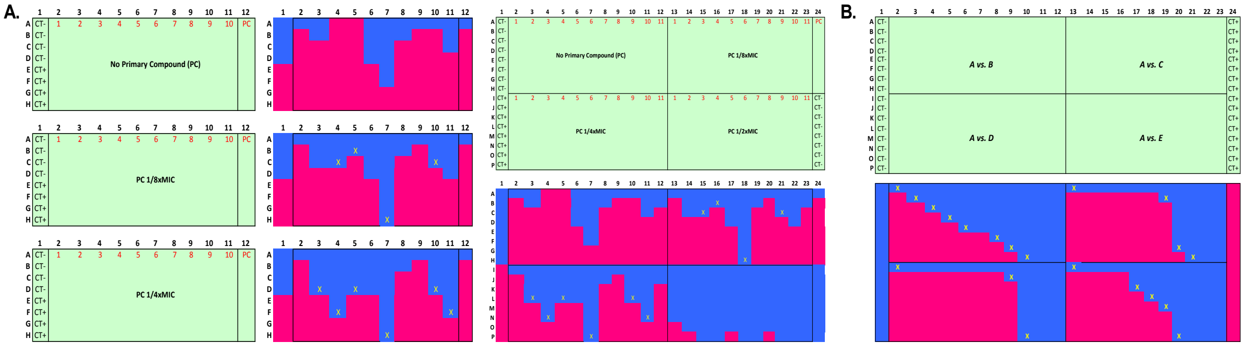

Figure S2. Rifampicin accumulation in *M. bovis* BGC. (A) Rifampicin (RIF) at 1 and 10 µg/mL was added to cell cultures incubated at 4 or 37˚C. A temperature-dependent active rifampicin accumulation profile was observed. Accumulation of rifampicin in cultures supplemented with 10 µg/mL did not yield proportional intracellular levels of rifampicin compared to the lower 1 µg/mL rifampicin concentration, indicating a saturated active uptake system. High dose rifampicin (10 µg/mL) reduced cell viability under the assay conditions. A low dose of 1 µg/mL rifampicin incubated at 37˚C was selected for further accumulation studies. (B) Cells were pre-treated overnight in the presence of different beta-lactam synergistic partners before the rifampicin accumulation assay. The presence of beta-lactams increased accumulation levels similar to those observed in the presence of ethambutol.
A, amoxicillin; E, ethambutol; F, faropenem; H, isoniazid; M, meropenem; R, rifampicin; V, clavulanate; X, cefadroxil.

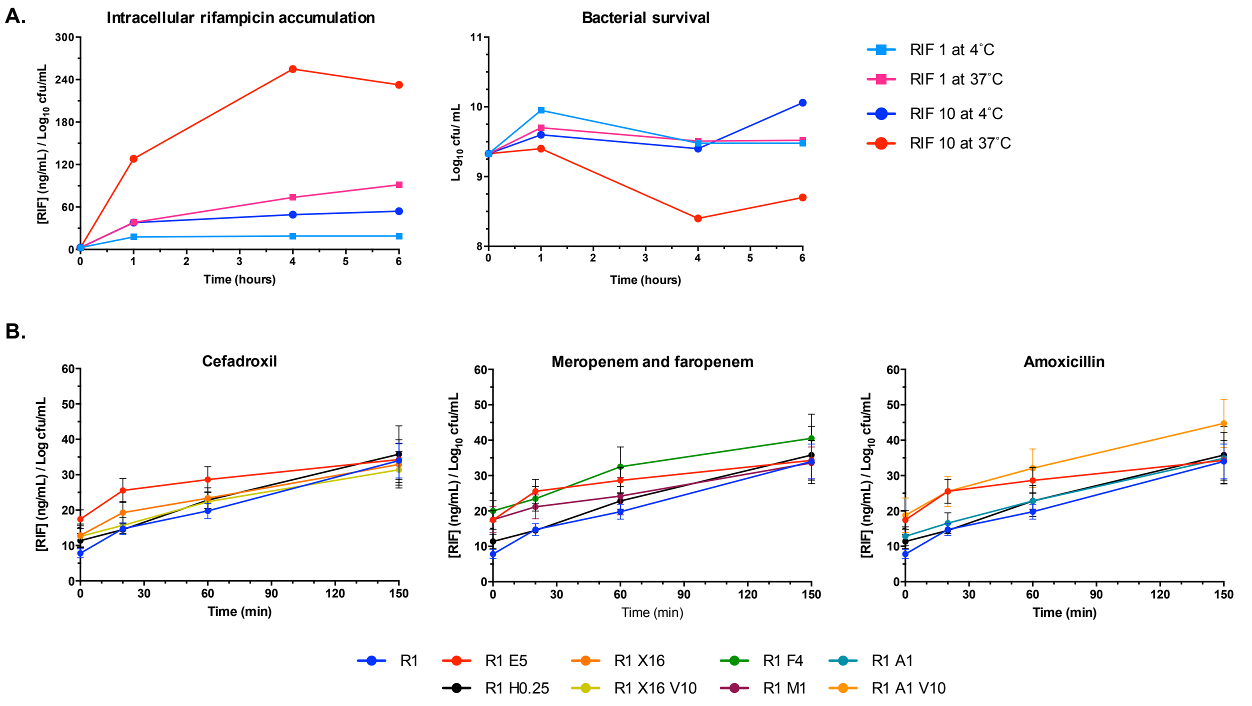

Figure S3. Kill kinetics of rifampicin plus cephradine alone and in combination against stationary *M. tuberculosis* H37Rv. MIC values of the individual drugs are used to express drug concentrations (i.e., 1x, 4x, 10x fold their MIC concentrations). RIF, rifampicin; CPD, cephradine. MIC_{RIF}= 0.03 µg/mL; MIC_{CPD}= 8 µg/mL.

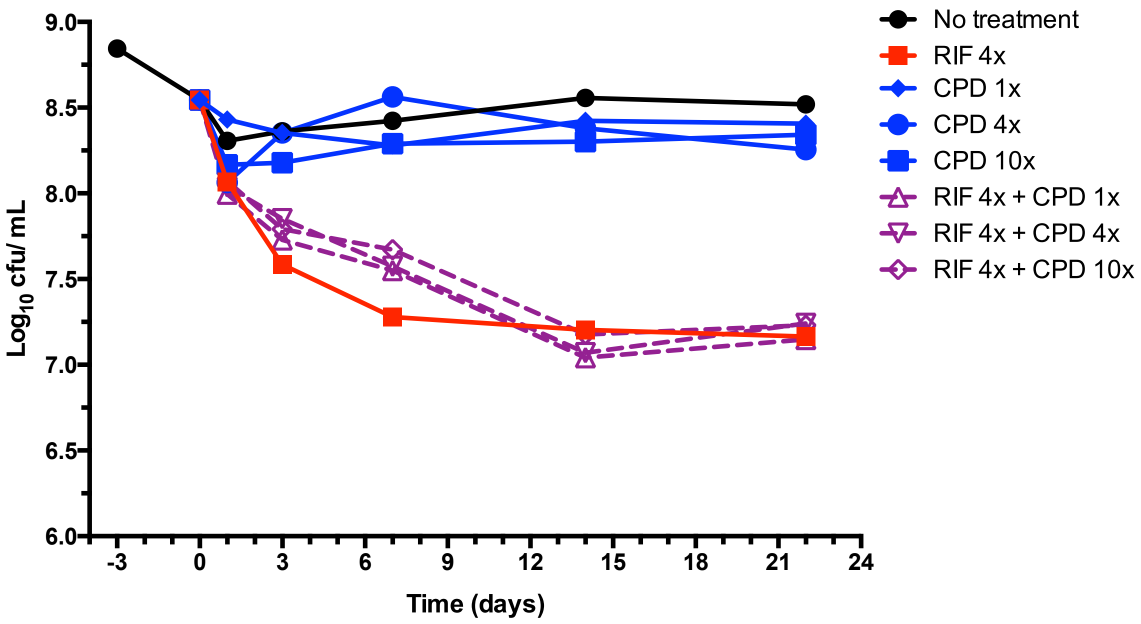

Figure S4. Synergistic triple combinations of rifampicin and beta-lactams. Dose response curves of rifampicin alone and in the presence of several beta-lactam combinations against (A) *M. tuberculosis* H37Rv (rifampicin susceptible) and (B) its rifampicin resistant derivative *M. tuberculosis* H37Rv H526D. For the drug susceptible strain, rifampicin, beta-lactam and clavulanate concentrations were 0.5 ng/mL, 0.06 µg/mL and 8 µg/mL, respectively. For the rifampicin resistant strain, rifampicin, beta-lactam and clavulanate concentrations were 8 µg/mL, 0.125 µg/mL and 5 µg/mL, respectively. Dose response curves of beta-lactams are expressed in µg/mL, as well as those of rifampicin for the resistant strains. Dose response curves of rifampicin for H37Rv are expressed in ng/mL. AMX, amoxicillin; CFX, cefadroxil; CLV, clavulanate; FAR, faropenem; MER, meropenem; and RIF, rifampicin.

A.

B.

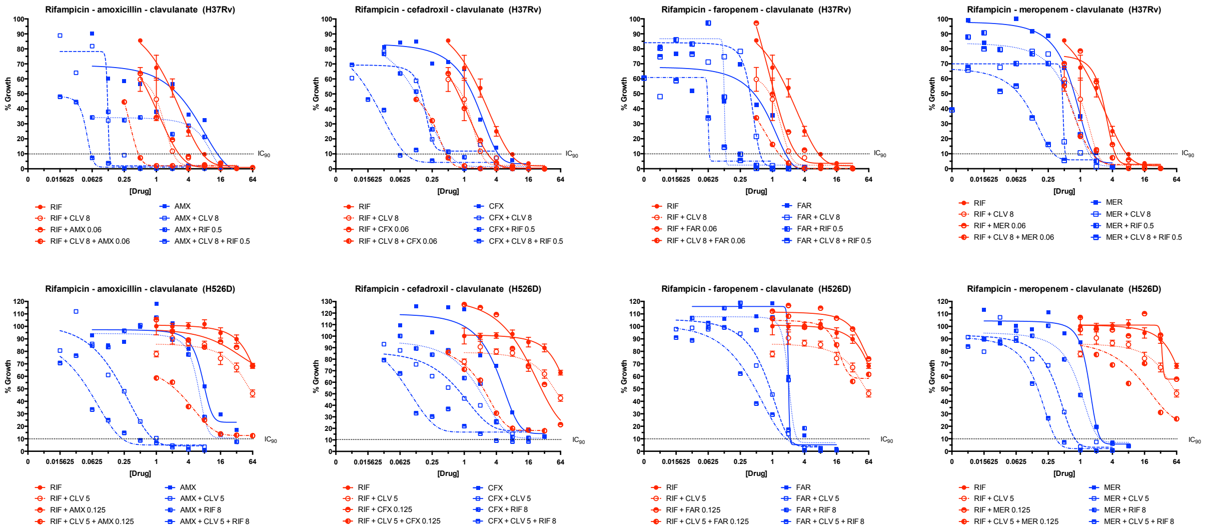

TABLE LEGENDS

Table S1. Strains and compounds used in this study.

Table S2. Rifampin and ethambutol synergy screen hits against *M. bovis* BCG. Hits were identified as those compounds whose MIC values decreased at least 4-fold in the presence of 1/4xMIC and/or 1/8xMIC concentrations of either rifampin or ethambutol. Hits were prioritized as follows: (i) a 4-fold MIC reduction in the “1/4xMIC” and/ or “1/8xMIC” group compared to “no primary compound” group; (ii) we excluded those compounds exhibiting MIC reductions higher than 128-fold or with fold-reduction ratios “1/4 vs. 1/8” higher than 64-fold (these were considered as artefacts); and finally, (iii) hits with escalating dose response, i.e. higher MIC fold reduction to untreated in the “1/4xMIC” group compared to the “1/8xMIC” group. MIC_{RIF} = 0.03125 µg/mL; MIC_{EMB} = 2 µg/mL. "400" values are assigned arbitrarily and not experimentally confirmed, only used for calculation purposes. Sy, synergy; P, potentiation; N.I., no interactions; Sy*, data from Ramón-García *et al.* (2011) AAC 55: 3861-3869.

Table S3. Extracellular and intracellular antimicrobial and synergistic activities of the cephalosporins and other cell wall targeting compounds against *Mycobacterium tuberculosis* strains, including multidrug resistant clinical isolates. BC strains are clinical isolates from British Columbia, Canada. DS, Drug sensitive; MDR, multi-drug resistant. FICI, Fractional Inhibitory Concentration Index of the RIF-CPH combination. FICI values ≤ 0.5 indicate synergism. RIF FICI range indicates the lowest and highest FICI of the combination against that particular set of strains. ROA, route of administration; po, oral; iv, intravenous; im, intramuscular; tp, topical.

Table S4. Activity-based qualitative classification of cephalosporins against *M. tuberculosis* H37Rv. Good activity (MIC ≤ 8 µg/mL); Moderate activity (MIC ≤ 16-64 µg/mL); Low activity (MIC ≥128 µg/mL). Cephalosporin generation as per established literature.
MIC against *M. tuberculosis* H37Rv
RIF FICI, synergistici interactions with rifampicin. FICI ≤0.5 indicates synergy.
Pan-MDR active, displays activity across drug susceptible and drug-resistant clinical isolates.

Table S5. Synergistic interactions between rifamycins, beta-lactams and other cell-wall targeting compounds. Fold reduction indicates the change in MIC of the beta-lactam in the presence of 1/4xMIC of the rifamycin compared to the MIC of the beta-lactam alone.
MIC_{RIF}= 16 ng/mL; MIC_{RPT}= 4 ng/mL; MIC_{RBT}= 2 ng/mL
RIF, rifampicin; RPT, rifapentine; RBT, rifabutin.

Table S6. Pharmacological properties of oral cephalosporins. Data from John Hopkins ABX guide: http://www.hopkinsguides.com/hopkins/index/Johns_Hopkins_ABX_Guide/All_Topics/A.

Table S7. Amoxicillin plus beta-lactams against *M. tuberculosis* H37Rv and *M. tuberculosis* H37Rv H526D (RIF resistant). Fold reduction indicates the change in MIC of the compound in the combination compared to the activity of the compound alone.
AMX, amoxicillin; CFX, cefadroxil; CLV, clavuanate; FAR, faropenem; MER, meropenem.
FICI, fractional inhibitory concentration index of the combination.
A FICI ≤ 0.5 indicates synergism. The closer the value to zero the stronger the synergistc effect of the combination.

Table S1. Strains and compounds used in this study.

**Strain**		**Drug resistance profile**
M. bovis	BCG Pasteur	WT
M. tuberculosis	H37Rv	WT (ATCC 25618)
M. tuberculosis	H37Rv-Luc	HYG
M. tuberculosis	H37Rv	RIF (H526D)
M. tuberculosis	CDC 1551	WT
M. tuberculosis	Erdman	WT
M. tuberculosis	BC-DS1	-
M. tuberculosis	BC-DS3	-
M. tuberculosis	BC-DS4	-
M. tuberculosis	BC-DS5	-
M. tuberculosis	BC-MDR2	INH, RIF, PZA, SM, RBT
M. tuberculosis	BC-MDR3	INH, RIF, RBT
M. tuberculosis	BC-MDR4	INH, RIF, EMB, PZA, RBT
M. tuberculosis	BC-MDR5	INH, RIF, PZA, SM, PAS, RBT
**Compounds**	**Provider**	
Rifamycins		
Rifabutin	LKT Laboratories, Inc. & Sigma-Aldrich	
Rifampicin	Chemos & Sigma-Aldrich	
Rifapentine	AK Scientific, Inc. & Sigma-Aldrich	
Cephalosporins		
Cefaclor	LKT Laboratories, Inc.	
Cefadroxil	MP Biologicals & GlaxoSmithKline	
Cefamandole	Sigma-Aldrich	
Cefapirin	Sigma-Aldrich & GlaxoSmithKline	
Cefazolin	Sigma-Aldrich	
Cefcapene pivoxil	Ochem Incorporation	
Cefdinir	Sigma-Aldrich & GlaxoSmithKline	
Cefditoren pivoxil	US Pharmacopeia & Waterstone Technologies, LLC	
Cefepime	AK Scientific, Inc.	
Cefixime	AK Scientific, Inc.	
Cefmetazole	Sigma-Aldrich	
Cefodizime	Sequoia	
Cefonicid sodium	Waterstone Technologies, LLC	
Cefoperazone	LKT Laboratories, Inc.	
Ceforanide	GlaxoSmithKline	
Cefotaxime	Sigma-Aldrich	
Cefotiam hydrochloride	Waterstone Technologies, LLC	
Cefoxitin	Sigma-Aldrich	
Cefpodoxime proxetil	Sequoia	
Cefprozil	AK Scientific, Inc.	
Ceftazidime	Sigma-Aldrich	
Ceftibuten	Sigma-Aldrich	
Ceftiofur	LKT Laboratories, Inc.	
Ceftriaxone	Sigma-Aldrich	
Cefuroxime	Sigma-Aldrich	
Cephalexin	Sigma-Aldrich & GlaxoSmithKline	
Cephalothin	Sigma-Aldrich	
Cephradine	Toku-e & GlaxoSmithKline	
Carbapenems		
Meropenem	Molekula & Sigma-Aldrich	
Penems		
Amoxicillin	Sigma-Aldrich	
Ampicillin	Sigma-Aldrich	
Faropenem	Sequoia	
Beta-lactam inhibitors		
Clavulanate	AK Scientific, Inc.	
Tazobactam	AK Scientific, Inc.	
Cell wall inhibitors		
Bacitracin	Sigma-Aldrich	
Ethambutol	MP Biologicals	
Isoniazid	Sigma-Aldrich	
Vancomycin	Sigma-Aldrich	
Others		
Amikacin	GlaxoSmithKline	
Bedaquiline	GlaxoSmithKline	
Clofazimine	GlaxoSmithKline	
Delamanid	GlaxoSmithKline	
Ethionamide	GlaxoSmithKline	
Hygromycin B	Sigma-Aldrich	
Levofloxacin	GlaxoSmithKline	
Linezolid	GlaxoSmithKline	
Moxifloxacin	GlaxoSmithKline	
p-Aminosalicilate	GlaxoSmithKline	
PA-824	GlaxoSmithKline	
SQ-109	GlaxoSmithKline	
Streptomycin	GlaxoSmithKline	
Thioridazine	GlaxoSmithKline	

Table S2. Rifampin and ethambutol synergy screen hits against *M. bovis* BCG.

						RIFAMPICIN HTSS							ETHAMBUTOL HTSS					
						MIC (µM) of compound in the presence of RIF at:			Fold change to untreated				MIC (µM) of compound in the presence of EMB at:			Fold change to untreated		
**Compound**	**Class**	**Bacterial Target**	**Clinical Indication**	**RIF hit (n=50)**	**EMB hit (n= 65)**	**0**	**1/8xMIC**	**1/4xMIC**	**1/8xMIC**	**1/4xMIC**	**[1/4]/[1/8]**	**BCG Validation - RIF FICI**	**0**	**1/8xMIC**	**1/4xMIC**	**1/8xMIC**	**1/4xMIC**	**[1/4]/[1/8]**
Tolfenamic acid			Anti-inflammatory	X		400	100	100	4	4	1		400	400	400	1	1	1
Sulbactam	Beta-lactamase inhibitor	Cell wall	Antibacterial	X		400	100	100	4	4	1		400	400	400	1	1	1
Biapenem	Carbapenem	Cell wall	Antibacterial	X	X	25	6.25	6.25	4	4	1		6.25	6.25	1.5625	1	4	4
Doripenem	Carbapenem	Cell wall	Antibacterial	X	X	6.25	6.25	1.5625	1	4	4		6.25	6.25	1.5625	1	4	4
Meropenem	Carbapenem	Cell wall	Antibacterial	X	X	100	100	25	1	4	4		100	25	25	4	4	1
Cefaclor	Cephalosporin	Cell wall	Antibacterial	X		400	400	100	1	4	4		400	400	400	1	1	1
Cefadroxil	Cephalosporin	Cell wall	Antibacterial	X		400	100	6.25	4	64	16		400	400	1.5625	1	256	256
Cefapirin	Cephalosporin	Cell wall	Antibacterial	X	X	100	25	25	4	4	1	0.125 (Sy)	100	25	6.25	4	16	4
Cefdinir	Cephalosporin	Cell wall	Antibacterial	X	X	100	100	25	1	4	4	0.15 (Sy)	100	100	25	1	4	4
Cefmetazole	Cephalosporin	Cell wall	Antibacterial		X	400	400	400	1	1	1		400	100	100	4	4	1
Cefotaxime	Cephalosporin	Cell wall	Antibacterial	X	X	400	25	25	16	16	1		100	25	25	4	4	1
Cefoxitin sodium	Cephalosporin	Cell wall	Antibacterial		X	400	400	400	1	1	1		400	400	100	1	4	4
Ceftazidime hydrate	Cephalosporin	Cell wall	Antibacterial		X	400	400	400	1	1	1		400	400	100	1	4	4
Ceftiofur	Cephalosporin	Cell wall	Antibacterial	X		400	400	100	1	4	4	0.375 (Sy)	400	400	400	1	1	1
Cephalexin	Cephalosporin	Cell wall	Antibacterial	X	X	400	100	100	4	4	1		400	400	25	1	16	16
Cephalothin sodium	Cephalosporin	Cell wall	Antibacterial		X	400	400	400	1	1	1		400	400	100	1	4	4
Cephradine	Cephalosporin	Cell wall	Antibacterial	X	X	400	100	6.25	4	64	16		400	400	6.25	1	64	64
Ampicillin	Penem	Cell wall	Antibacterial	X	X	400	100	100	4	4	1		400	100	100	4	4	1
Ethambutol		Cell wall	Antibacterial	X	NA	25	25	6.25	1	4	4	0.375 (Sy)	NA	NA	NA	NA	NA	NA
Balofloxacin	Quinolone	DNA	Antibacterial		X	1.5625	1.5625	1.5625	1	1	1	1.0 (N.I.)	1.5625	1.5625	0.3906	1	4	4
Fleroxacin	Quinolone	DNA	Antibacterial	X	X	25	25	6.25	1	4	4		25	25	6.25	1	4	4
Gatifloxacin	Quinolone	DNA	Antibacterial	X	X	0.3906	0.3906	0.0977	1	4	4		0.3906	0.3906	0.0977	1	4	4
Pefloxacin	Quinolone	DNA	Antibacterial	X	X	100	25	25	4	4	1		100	25	25	4	4	1
Sulfadimethoxine	Sulfonamide	Folate synthesis	Antibacterial	X		400	400	100	1	4	4		400	400	400	1	1	1
Sulfaquinoxaline	Sulfonamide	Folate synthesis	Antibacterial		X	100	100	100	1	1	1		100	100	25	1	4	4
Sulfathiazole	Sulfonamide	Folate synthesis	Antibacterial		X	25	25	25	1	1	1		100	25	25	4	4	1
Carbonyl Cyanide m-Chlorophenyl hydrazon		Ionophore	Antibacterial	X	X	100	25	25	4	4	1		25	25	6.25	1	4	4
Nigericin		Ionophore	Antibacterial	X		1.5625	0.3906	0.3906	4	4	1		1.5625	1.5625	1.5625	1	1	1
Salinomycin		Ionophore	Antibacterial	X	X	25	25	6.25	1	4	4		25	6.25	1.5625	4	16	4
Thiacetazone		Membrane	Antibacterial	X		100	25	6.25	4	16	4	0.31 (Sy)	100	100	100	1	1	1
Clofazimine	Riminophenazine	Multiple	Antibacterial	X	X	6.25	1.5625	1.5625	4	4	1		6.25	1.5625	1.5625	4	4	1
Nitrofurantoin		Multiple	Antibacterial	X	X	400	25	25	16	16	1	0.56 (P)	400	100	25	4	16	4
Neomycin	Aminoglycoside	Ribosome	Antibacterial	X		400	400	100	1	4	4		400	100	400	4	1	0.25
Streptomycin	Aminoglycoside	Ribosome	Antibacterial	X		0.3906	0.3906	0.0977	1	4	4		0.0977	0.0977	0.0977	1	1	1
Tobramycin	Aminoglycoside	Ribosome	Antibacterial	X	X	400	25	25	16	16	1	1.0 (N.I.)	400	25	25	16	16	1
Clindamycin	Lincosamide	Ribosome	Antibacterial		X	6.25	6.25	6.25	1	1	1		6.25	6.25	1.5625	1	4	4
Midecamycin	Macrolide	Ribosome	Antibacterial		X	400	400	400	1	1	1		400	400	25	1	16	16
Roxithromycin	Macrolide	Ribosome	Antibacterial	X		25	25	6.25	1	4	4		25	25	0.0244	1	1024	1024
Spiramycin	Macrolide	Ribosome	Antibacterial		X	400	400	400	1	1	1		400	400	6.25	1	64	64
Tylosin	Macrolide	Ribosome	Antibacterial	X	X	400	25	25	16	16	1		400	25	25	16	16	1
Anhydrotetracycline	Tetracycline	Ribosome	Antibacterial		X	400	400	400	1	1	1		400	100	100	4	4	1
Chlortetracycline	Tetracycline	Ribosome	Antibacterial		X	25	25	25	1	1	1		25	25	6.25	1	4	4
Meclocycline	Tetracycline	Ribosome	Antibacterial	X	X	25	6.25	6.25	4	4	1		25	6.25	6.25	4	4	1
Methacycline	Tetracycline	Ribosome	Antibacterial	X		400	25	25	16	16	1		400	400	400	1	1	1
Puromycin		Ribosome	Antibacterial	X	X	25	6.25	6.25	4	4	1		25	6.25	6.25	4	4	1
Naphthomycin B	Ansamycin	RNAP	Antibacterial		X	100	100	100	1	1	1		400	100	100	4	4	1
Rifampicin	Rifamycin	RNAP	Antibacterial	NA	X	NA	NA	NA	NA	NA	NA		0.0977	0.0244	0.0244	4	4	1
Rifaximine	Rifamycin	RNAP	Antibacterial		X	NA	NA	NA	NA	NA	NA		0.0244	0.0061	0.0061	4	4	1
Rabeprazole		Proton pump inhibitor	Anticancer		X	100	400	400	0.25	0.25	1		100	100	25	1	4	4
Geldanamycin	Ansamycin	RNAP	Anticancer	X		100	25	25	4	4	1		100	100	100	1	1	1
beta-Lapachone			Anticancer	X	X	100	100	25	1	4	4		100	100	25	1	4	4
Tamoxifen			Anticancer		X	100	100	100	1	1	1		100	25	25	4	4	1
Itraconazole	Azole		Antifungal		X	400	400	400	1	1	1		400	400	25	1	16	16
Ketoconazole	Azole		Antifungal	X	X	100	25	25	4	4	1	Sy*	100	100	25	1	4	4
Miconazole	Azole		Antifungal		X	25	25	25	1	1	1		25	6.25	6.25	4	4	1
Ornidazole	Azole		Antiprotozoal		X	100	100	100	1	1	1		100	100	6.25	1	16	16
Sertaconazole	Azole		Antifungal	X	X	100	100	25	1	4	4		100	25	25	4	4	1
Terconazole	Azole		Antifungal		X	100	100	100	1	1	1		100	25	25	4	4	1
Mebendazole	Benzimidazole		Antifungal	X		100	25	25	4	4	1		25	25	25	1	1	1
Chlorhexidine			Antifungal	X		25	6.25	6.25	4	4	1		25	6.25	25	4	1	0.25
Clioquinol			Antifungal	X		25	6.25	6.25	4	4	1		25	25	25	1	1	1
Phenylmercuric acetate			Antifungal	X	X	400	6.25	6.25	64	64	1		400	6.25	6.25	64	64	1
Thimerosal			Antifungal		X	6.25	25	6.25	0.25	1	4		6.25	1.5625	1.5625	4	4	1
Terfenadine			Antihistamine		X	25	25	25	1	1	1		25	6.25	6.25	4	4	1
Doramectin	Avermectin		Antiparasitic	NA	X	NA	NA	NA					25	25	6.25	1	4	4
Moxidectin	Avermectin		Antiparasitic		X	25	25	25	1	1	1		25	25	6.25	1	4	4
Maduramicin		Ionophore	Antiprotozoal	X	X	400	25	25	16	16	1		400	6.25	6.25	64	64	1
Bromperidol	Butyrophenone		Antipsychotic	X	X	400	400	100	1	4	4	Sy*	400	100	100	4	4	1
Trifluperidol	Butyrophenone		Antipsychotic	X	X	400	100	100	4	4	1		400	100	25	4	16	4
Prochlorperazine	Phenothiazine		Antipsychotic		X	1.5625	0.3906	1.5625	4	1	0.25		1.5625	1.5625	0.3906	1	4	4
Trifluoperizine	Phenothiazine		Antipsychotic	X	X	400	25	25	16	16	1		400	25	6.25	16	64	4
Aripiprazole			Antipsychotic		X	400	25	100	16	4	0.25		400	100	25	4	16	4
Ethacridine lactate		DNA	Antiseptic	X	X	25	25	6.25	1	4	4		25	25	6.25	1	4	4
Spinosad (Spinosyn A and Spinosyn D)			Insecticide	X		400	400	100	1	4	4		400	400	400	1	1	1
Diacylglycerol Kinase Inhibitor II			Kinase inhibitor		X	400	400	400	1	1	1		400	100	100	4	4	1
Imatinib			Kinase inhibitor		X	400	400	400	1	1	1		400	100	100	4	4	1
Stigmatellin			Mitochondria		X	400	400	400	1	1	1		400	25	1.5625	16	256	16
BAY 11-7085			NFkB		X	100	100	100	1	1	1		100	100	25	1	4	4
Cerulenin		Cell wall		NA	X	NA	NA	NA					6.25	1.5625	1.5625	4	4	1
Valinomycin	Peptide	Ionophore			X	0.0244	0.0244	0.0244	1	1	1	0.56 (P)	0.0244	0.0061	0.0061	4	4	1
Totarol	Terpene			X		400	400	100	1	4	4		400	400	100	1	4	4
Fiduxosin					X	100	100	100	1	1	1		100	25	6.25	4	16	4
Oleanolic acid				X	X	400	400	100	1	4	4		400	400	100	1	4	4
Thiourea				X	X	400	100	100	4	4	1		400	100	100	4	4	1

Table S3. Extracellular and intracellular antimicrobial and synergistic activities of the cephalosporins and other cell wall targeting compounds against *Mycobacterium tuberculosis* strains, including multidrug resistant clinical isolates.

			Extracellular MIC (µg/mL) for *Mycobacterium tuberculosis* strains:											RIF FICI range			Intracellular (THP1 H37Rv-Luc)		THP1 toxicity
**Compound**	**ROA**	**Generation**	**H37Rv**	**CDC 1551**	**Erdman**	**BC-DS1**	**BC-DS3**	**BC-DS4**	**BC-DS5**	**BC-MDR2**	**BC-MDR3**	**BC-MDR4**	**BC-MDR5**	**H37Rv**	**BC-DS**	**BC-MDR**	**MIC (µg/mL)**	**RIF FICI**	**IC50 (µg/mL)**
Cephems																			
Cefadroxil	po	1st	4-8	4-8	8	<0.25	4	0.5-1	4	8-16	16	8	32	0.18	nd	nd	64	0.19	>64
Cefapirin	iv	1st	2	2	1-2	2	8	2	16	16	8	4	8	0.18 - 0.37	nd	nd	>8	nd	nd
Cefazolin	iv, im	1st	64	32-64	32	64-128	>128	64	>128	>128	>128	128	>128	nd	nd	nd	>32	nd	nd
Cephalexin	po	1st	8-16	8-16	16	1	8	2	16	16-32	32	16	64-128	0.12 - 0.18	0.18 - 0.31	0.09 - 0.28	>32	0.39	nd
Cephalothin	iv	1st	32-64	32	16-32	64-128	128-256	64	>128	>128	>128	64-128	>128	nd	nd	nd	nd	nd	nd
Cephradine	po, iv	1st	4-8	4	8	0.5	2-4	0.5-1	4	8	8-16	4	16-32	0.18	0.18 - 0.37	0.09 - 0.28	32 - >32	0.39	nd
Cefaclor	po	2nd	128	128	128	32	128	64	128	128	128	128	>128	nd	nd	nd	nd	nd	nd
Cefamandole	iv	2nd	>128	>128	>128	>128	>128	>128	>128	>!28	>128	>128	>128	nd	nd	nd	>32	nd	nd
Cefmetazole	iv	2nd	64-128	32-64	16	128	>128	64	>128	>128	>128	64	64	nd	nd	nd	nd	nd	nd
Cefonicid	iv, im	2nd	>32	nd	nd	nd	nd	nd	nd	nd	nd	nd	nd	nd	nd	nd	>32	nd	nd
Ceforanide	iv	2nd	2	nd	nd	nd	nd	nd	nd	nd	nd	nd	nd	nd	nd	nd	nd	nd	nd
Cefotiam	iv	2nd	>32	nd	nd	nd	nd	nd	nd	nd	nd	nd	nd	nd	nd	nd	>32	nd	nd
Cefoxitin	iv	2nd	>128	128	64-128	>128	>128	>128	>128	>128	>128	>128	>128	nd	nd	nd	>32	nd	nd
Cefprozil	po	2nd	4-8	8	8-16	4	16	2-4	128	16-32	>64	32	>64	0.31 - 0.37	nd	nd	nd	nd	nd
Cefuroxime	po, iv	2nd	32	16-32	16	64	128	32	>128	>128	>128	32	128	0.5 - 0.56	nd	nd	nd	nd	nd
Cefcapene pivoxil	po	3rd	>32	nd	nd	nd	nd	nd	nd	nd	nd	nd	nd	nd	nd	nd	>32	nd	nd
Cefdinir	po	3rd	1-2	2	1-2	2-4	8	2	16	16	8	4	8	0.31 - 0.37	nd	nd	2-8	0.19	>64
Cefditoren pivoxil	po	3rd	128	64	64	128-256	>128	64-128	>128	>128	>128	128	>128	0.625	nd	nd	>32	nd	nd
Cefixime	po	3rd	128	64-128	128	64	128	32	>128	>128	>128	64	>128	nd	nd	nd	>32	nd	nd
Cefodizime	iv, im	3rd	32	16-32	16	32	128	32	>128	>128	>128	16	64-128	nd	nd	nd	nd	nd	nd
Cefoperazone	iv	3rd	>128	>128	>128	>128	>128	>128	>128	>128	>128	>128	>128	nd	nd	nd	nd	nd	nd
Cefotaxime	iv	3rd	4-8	4-8	8	4	16	4	32	16-32	16	4	16	nd	nd	nd	nd	nd	nd
Cefpodoxime proxetil	po	3rd	>128	>128	>128	128	128	32-64	>128	128	>128	128	>128	nd	nd	nd	nd	nd	nd
Ceftazidime	iv, im	3rd	>128	>128	>128	>128	>128	>128	>128	>128	>128	>128	>128	nd	nd	nd	nd	nd	nd
Ceftibuten	po	3rd	>128	>128	>128	>128	>128	>128	>128	>128	>128	>128	>128	0.62 - 0.75	nd	nd	nd	nd	nd
Ceftiofur		3rd	8-16	8	4-8	16	32	8-16	128	32-64	64	8	32	0.37 - 0.5	nd	nd	nd	nd	nd
Ceftriaxone	iv	3rd	16-32	16	8	16	64	16	>128	128	128	16	64	nd	nd	nd	nd	nd	nd
Cefepime	iv	4th	2-4	4	4	2	8	2-4	16-32	16	8	8	16	nd	nd	nd	nd	nd	nd
Carbapenems																			
Meropenem	iv		1-2	2-4	2-4	2	16	2	32	>!6	16	16	16	0.37 - 0.5	nd	nd	16-32	1.0	>64
Penems																			
Amoxicillin	po		16-32	16-32	16	32	>64	32	>64	>64	64	64	>64	0.375	nd	nd	>32	nd	>64
Ampicillin	po		>128	nd	nd	nd	nd	nd	nd	nd	nd	nd	nd	0.375	nd	nd	nd	nd	nd
Faropenem	po		4	4	2	4	8	2	16	8	8	4	4	0.25-0.375	nd	nd	16-32	0.25	>64
Beta-lactam inhibitors																			
Clavulanate	po		64-128	64-128	128	32	64	32	32-64	64	64-128	64	64-128	0.18 - 0.25	nd	nd	>32	nd	nd
Tazobactam	po		64-128	64	64-128	32	128	32	64	>128	>128	128	>128	0.18 - 0.25	nd	nd	nd	nd	nd
Other cell wall inhibitors																			
Bacitracin	tp		128	nd	nd	nd	nd	nd	nd	nd	nd	nd	nd	2	nd	nd	nd	nd	nd
Ethambutol	po		1-4	nd	nd	4	2	2	4	4	2	32	nd	0.26 - 0.375	nd	nd	>32	0.19	nd
Isoniazid	po		0.05	nd	nd	nd	nd	nd	nd	nd	nd	nd	nd	2	nd	nd	nd	nd	nd
Vancomycin	po		16	nd	nd	nd	nd	nd	nd	nd	nd	nd	nd	1-2	nd	nd	nd	nd	nd

BC strains are clinical isolates from British Columbia, Canada. DS, Drug sensitive; MDR, multi-drug resistant.
FICI, Fractional Inhibitory Concentration Index of the RIF-CPH combination. FICI values ≤ 0.5 indicate synergism.
RIF FICI range indicates the lowest and highest FICI of the combination against that particular set of strains.\
ROA, route of administration; po, oral; iv, intravenous; im, intramuscular; tp, topical.

Table S4. Activity-based qualitative classification of cephalosporins against *M. tuberculosis* H37Rv

Cephalosporin	Generation	MIC (µg/mL)	RIF FICI	Pan-MDR active	Smile
Good activity					
Cefdinir	3rd	1-2	0.31-0.37	Yes	[H][C@]12SCC(C=C)=C(N1C(=O)[C@H]2NC(=O)C(=N/O)\C1=CSC(N)=N1)C(O)=O
Cefapirin	1st	2	0.18 - 0.37	Yes	O=C(C(N12)=C(COC(C)=O)CS[C@]2([H])[C@H](NC(CSC3=CC=NC=C3)=O)C1=O)[O-].[Na+]
Cefepime	4th	2-4	nd	Yes	O=C(C(N12)=C(C[N+]3(C)CCCC3)CS[C@]2([H])[C@H](NC(/C(C4=CSC(N)=N4)=N\OC)=O)C1=O)[O-]
Cefotaxime	3rd	4-8	nd	Yes	O=C(C(N12)=C(COC(C)=O)CS[C@]2([H])[C@H](NC(/C(C3=CSC(N)=N3)=N\OC)=O)C1=O)O
Cefadroxil	1st	8	0.18	Yes	[H][C@]12SCC(C)=C(N1C(=O)[C@H]2NC(=O)[C@H](N)C1=CC=C(O)C=C1)C(O)=O
Cephradine	1st	8	0.18	Yes	O=C(C(N12)=C(C)CS[C@]2([H])[C@H](NC([C@H](N)C3=CCC=CC3)=O)C1=O)O
Cefprozil	2nd	8	0.31-0.37	No	O=C(C(N12)=C(/C=C/C)CS[C@]2([H])[C@H](NC([C@H](N)C3=CC=C(O)C=C3)=O)C1=O)O
Moderate activity					
Cephalexin	1st	16	0.12-0.18	No	O=C(C(N12)=C(C)CS[C@]2([H])[C@H](NC([C@H](N)C3=CC=CC=C3)=O)C1=O)O
Ceftiofur	3rd	8-16	0.37-0.5	No	O=C(C(N12)=C(CSC(C3=CC=CO3)=O)CS[C@]2([H])[C@H](NC(/C(C4=CSC(N)=N4)=N\OC)=O)C1=O)O
Ceftriaxone	3rd	16-32	nd	No	O=C(C(N12)=C(CSC(N(C)NC3=O)=NC3=O)CS[C@]2([H])[C@H](/N=C(O)\C(C4=CSC(N4)=N)=N/OC)C1=O)O
Cefodizime	3rd	32	nd	No	O=C(C(N12)=C(CSC3=NC(C)=C(CC(O)=O)S3)CS[C@]2([H])[C@H](NC(/C(C4=CSC(N)=N4)=N\OC)=O)C1=O)O
Cefuroxime	2nd	32	0.5-0.56	No	O=C(C(N12)=C(COC(N)=O)CS[C@]2([H])[C@H](NC(/C(C3=CC=CO3)=N\OC)=O)C1=O)O
Cefazolin	1st	64	nd	No	[H][C@]12SCC(CSC3=NN=C(C)S3)=C(N1C(=O)[C@H]2NC(=O)CN1C=NN=N1)C(O)=O
Cephalothin	1st	64	nd	No	O=C(C(N12)=C(COC(C)=O)CS[C@]2([H])[C@H](NC(CC3=CC=CS3)=O)C1=O)O
Low activity					
Cefaclor	2nd	128	nd	No	O=C(C(N12)=C(Cl)CS[C@]2([H])[C@H](NC([C@H](N)C3=CC=CC=C3)=O)C1=O)O
Cefmetazole	2nd	128	nd	No	O=C(C(N12)=C(CSC3=NN=NN3C)CS[C@]2([H])[C@](OC)(NC(CSCC#N)=O)C1=O)O
Cefditoren pivoxil	3rd	128	0.625	No	O=C(C(N12)=C(/C=C/C3=C(C)N=CS3)CS[C@]2([H])[C@H](NC(/C(C4=CSC(N)=N4)=N/OC)=O)C1=O)OCOC(C(C)(C)C)=O
Cefixime	3rd	128	nd	No	O=C(C(N12)=C(C=C)CS[C@]2([H])[C@H](NC(/C(C3=CSC(N)=N3)=N\OCC(O)=O)=O)C1=O)O
Cefocinid	2nd	>32	nd	nd	O=C(C(N12)=C(CSC3=NN=NN3CS(=O)(O)=O)CS[C@]2([H])[C@H](NC([C@H](O)C4=CC=CC=C4)=O)C1=O)O
Cefotiam	2nd	>32	nd	nd	O=C(C(N12)=C(CSC3=NN=NN3CCN(C)C)CS[C@]2([H])[C@H](NC(CC4=CSC(N)=N4)=O)C1=O)O
Cefamandole	2nd	>32	nd	No	O=C(C(N12)=C(CSC3=NN=NN3C)CS[C@]2([H])[C@H](NC([C@H](O)C4=CC=CC=C4)=O)C1=O)O

Good activity (MIC ≤ 8 µg/mL); Moderate activity (MIC ≤ 16-64 µg/mL); Low activity (MIC ≥128 µg/mL).

Cephalosporin generation as per established literature.

MIC against *M. tuberculosis* H37Rv

RIF FICI, synergistici interactions with rifampicin. FICI ≤0.5 indicates synergy.

Pan-MDR active, displays activity across drug susceptible and drug-resistant clinical isolates.

Table S5. Synergistic interactions between rifamycins, beta-lactams and other cell-wall targeting compounds.

	MIC (μg/mL) for *M. tuberculosis* H37Rv-Luc:										
	Amoxicillin	Cefadroxil	Cefamandole	Cefapirin	Cefazolin	Cefdinir	Cephradine	Ethambutol	Faropenem	Isoniazid	Meropenem
	16-32	8-16	32	2	16	2	16-32	2	2-4	0.25	4
Rifampicin											
1/4xMIC RIF	4	<0.125	8	0.5	4	0.25	<0.25	0.5	0.25	0.25	1
Fold reduction	4-8	>64	4	4	4	8	>64	4	8-16	1	4
Rifapentine											
1/4xMIC RPT	16	1	16	1	16	0.5	4	0.5	0.5	0.25	2
Fold reduction	1-2	8-16	2	2	1	4	4-8	4	4-8	1	2
Rifabutin											
1/4xMIC RBT	16	16	32	2	32	2	16	2	4	0.25	4
Fold reduction	1-2	0.5-1	1	1	0.5	1	1-2	1	0.5-1	1	1

Fold reduction indicates the change in MIC of the beta-lactam in the presence of 1/4xMIC of the rifamycin compared to the MIC of the beta-lactam alone.
MIC_RIF= 16 ng/mL; MIC_RPT= 4 ng/mL; MIC_RBT= 2 ng/mL
RIF, rifampicin; RPT, rifapentine; RBT, rifabutin

Table S6. Pharmacological properties of oral cephalosporins

Compound	ROA	Cmax	T1/2 (hrs)	Absorption (%)	Prot. Binding (%)	Comments
Cefaclor	PO	8-9 µg/ml after 500mg PO dose administration.	0.8	93	25-50	This drug is a favorite for pediatricians because it wins the taste test; safe to use during pregnancy; compatible with breast feeding. Diffuses readily into soft tissue interstitial fluid. Its concentration in sputum is usually low.
Cefadroxil	PO	16 µg/ml after 500mg PO dose administration.	1.5	90	20	Oral 1st generation cephalosporin with good oral bioavailability and long half-life allowing for once or twice a day dosing, but more expensive than comparable agents (e.g., cephalexin). Widely distributed to tissues and fluids. Low CNS penetration.
Cefdinir	PO	1.60 µg/ml after 300 mg dose; 2.87 µg/ml after 600 mg dose.	1.7	20-25	60-73	Oral 3rd generation cephalosporin that has activity against many gram-negative organisms, but is not active against Enterobacter and Pseudomonas. Activity against gram-positive bacteria is good and is similar to cefpodoxime. Distributed to tissues and fluids.
Cefditoren	PO	1.8 µg/ml (after 200mg) administer with or without food.	1.6+/- 0.4	Absolute bioavailability 16%. Food increases absorption by 70%.	88	Oral 3rd generation cephalosporin with spectrum of activity similar to cefpodoxime and cefdinir. A short half-life makes a twice a day dosing a concern for severe infections.
Cefixime	PO	3-5 µg/ml after400 mg PO dose administration.	3.1	30-50	65	Oral 3rd generation cephalosporin. Not well studied, but distributed in gallbladder, tonsillar, maxillary sinus tissue, middle ear and prostatic fluid. Poor penetration into Waldeyer's ring.
Cefpodoxime proxetil	PO	3 µg/ml after 200 mg PO dose administration.	2-3	46% absorbed. Absolute bioavailability ~50%. Food increases absorption by 21 to 33%.	40	Oral 3rd generation cephalosporin. More extensive clinical data exists with cefixime. Distributed to tissues and fluids.
Cefprozil	PO	10.5 µg/ml after 500 mg PO dose administration.	1.3-1.8	95	65	Oral 2nd generation cephalosporin with good activity against S. pneumoniae. One of the IDSA-ATS recommended 2nd generation cephalosporin for the treatment of CAP caused by PCN-susceptible S. pneumoniae, but not FDA-indicated. Distributed to tissues and fluids. Poor CNS penetration.
Ceftibuten	PO	15 µg/ml after 400 mg PO dose administration.	2.4	Liquid preparation must be administered on an empty stomach.	65	Oral 3rd generation cephalosporin. Not hydrolyzed by some extended-spectrum beta-lactamases. Distributed to tissues and fluids. Poor CNS penetration.
Cefuroxime	PO, IV	100 µg/ml after 1.5 Gm IV dose administration; 4 µg/ml after 250 mg PO dose.	1.5	52% oral absorption. Food increases absorption.	30-50	2nd generation oral and parenteral cephalosporin with convenient twice a day dosing schedule. Widely distributed to tissues and fluids.
Cephalexin	PO	18-38 µg/ml after 500 mg PO dose administration	1	90	5-15	Well absorbed 1st generation cephalosporin with good gram positive coverage and a low price but q6-8h dosing may decrease patient compliance. Widely distributed to tissues and fluids. Low CNS penetration.
Cephradine	PO, IV	86 µg/ml after 1 Gm IV dose administration. 16 µg/ml after 500 mg PO dose administration.	1.3	90	5-20	1st generation cephalosporin with similar spectrum of activity to cephalexin.Widely distributed to tissues and fluids. Poor CNS penetration. Well-absorbed cephalosporin giving similar serum concentration compared to IM administration. No longer available in the U.S.

Data from John Hopkins ABX guide.
http://www.hopkinsguides.com/hopkins/index/Johns_Hopkins_ABX_Guide/All_Topics/A (Date of access: 11/08/2016)

Table S7. Amoxicillin plus beta-lactams against M. tuberculosis H37Rv and M. tuberculosis H37Rv H526D (RIF resistant)

Drug(s) in combo		MIC (µg/mL) for M. tuberculosis H37Rv of:								FICI
**#1**	**#2**	**AMX**	**Fold**	**CFX**	**Fold**	**FAR**	**Fold**	**MER**	**Fold**	**Double**
Drugs alone		16	---	8	---	2	---	ND	---	---
AMX	CFX	2	8	0.5	16	---	---	---	---	0.19
AMX	FAR	2	8	---	---	0.5	4	---	---	0.38
		**MIC (µg/mL) for M. tuberculosis H37Rv H256D (RIFr) of:**								
Drugs alone		64	---	32	---	4	---	4	---	---
AMX	CFX	4	16	1	32	---	---	---	---	0.09
AMX	FAR	16	4	---	---	2	2	---	---	0.75
AMX	MER	2	32	---	---	---	---	1	4	0.28

Fold reduction indicates the change in MIC of the compound in the combination compared to the activity of the compound alone.
AMX, amoxicillin; CFX, cefadroxil; CLV, clavuanate; FAR, faropenem; MER, meropenem.
FICI, fractional inhibitory concentration index of the combination.
A FICI ≤ 0.5 indicates synergism. The closer the value to zero the stronger the synergistc effect of the combination.
